# Ultra-fast local-haplotype variant calling using paired-end DNA-sequencing data reveals somatic mosaicism in tumor and normal blood samples

**DOI:** 10.1093/nar/gkv953

**Published:** 2015-09-29

**Authors:** Subhajit Sengupta, Kamalakar Gulukota, Yitan Zhu, Carole Ober, Katherine Naughton, William Wentworth-Sheilds, Yuan Ji

**Affiliations:** 1 Program of Computational Genomics & Medicine, NorthShore University HealthSystem, Evanston, IL 60201, USA; 2 Center for Molecular Medicine, NorthShore University HealthSystem, Evanston, IL 60201, USA; 3 Department of Human Genetics, University of Chicago, Chicago, IL 60637, USA; 4 Department of Health Studies, University of Chicago, Chicago, IL 60637, USA

## Abstract

Somatic mosaicism refers to the existence of somatic mutations in a fraction of somatic cells in a single biological sample. Its importance has mainly been discussed in theory although experimental work has started to emerge linking somatic mosaicism to disease diagnosis. Through novel statistical modeling of paired-end DNA-sequencing data using blood-derived DNA from healthy donors as well as DNA from tumor samples, we present an ultra-fast computational pipeline, *LocHap* that searches for multiple single nucleotide variants (SNVs) that are scaffolded by the same reads. We refer to scaffolded SNVs as local haplotypes (LH). When an LH exhibits more than two genotypes, we call it a local haplotype variant (LHV). The presence of LHVs is considered evidence of somatic mosaicism because a genetically homogeneous cell population will not harbor LHVs. Applying LocHap to whole-genome and whole-exome sequence data in DNA from normal blood and tumor samples, we find wide-spread LHVs across the genome. Importantly, we find more LHVs in tumor samples than in normal samples, and more in older adults than in younger ones. We confirm the existence of LHVs and somatic mosaicism by validation studies in normal blood samples. LocHap is publicly available at http://www.compgenome.org/lochap.

## INTRODUCTION

Many cancers arise from a series of mutational events occurring throughout a person's life span (1,2). Considerable evidence (3,4) has accumulated supporting the presence of genetically heterogeneous cells in a somatic sample, a phenomenon called somatic mosaicism, which may be a precursor to the onset of many cancers (5). However, there are no effective and economical tools that can reliably measure the presence and degree of somatic mosaicism in a biological sample. Single cell sequencing (6) in principle provides the genetic landscape of each individual cells, although in practice only up to hundreds or thousands of cells can be measured due to the formidable cost of money and effort. In contrast, next-generation sequencing (NGS) technologies assemble an average genome sequence of all the cells in a sample, assuming cellular homogeneity. In the presence of somatic mosaicism, the average genome may not be a good representation of the sample. Despite continuous breakthroughs in DNA sequencing since the completion of the human genome project (7), researchers are still unable to precisely dissect individual cellular genomes on large scales.

Somatic mosaicism is often seen in samples derived from patients with cancer. Future targeted and personalized cancer therapy must take into account mosaic tumor cells in order to better customize therapies (8,9). In contrast, somatic mosaicism in samples from healthy individuals has been discussed as a theory over the last decade (10–12), with only a few recently reported examples (5,13–20). Due to the availability of high-throughput DNA sequencing, hundreds of millions of short reads can now be mapped to cover whole genomes or exomes. If somatic mosaicism is present in a biological sample, the DNA sequences of the short reads are expected to reflect the variations of the cellular genomes at the single nucleotide level. Based on this concept, pioneering work in 2014 by Genovese *et* *al*. (5) reported the presence of somatic mutations in blood samples as precursor of hematologic cancer and death. They carefully constructed bioinformatics and statistical methods to filter single nucleotide variants (SNVs) based on whole-exome and whole-genome sequencing data and identified clonal somatic blood samples with somatic mutations. Because the somatic mutations were only present in a fraction of the cells, the blood sample was considered mosaic. Their main computational analysis aimed to identify SNVs with variant allele fractions (VAFs) that are far <0.5 and attributed these SNVs to the existence of small cellular subpopulations harboring the SNVs. Computationally it is challenging to differentiate true biological subpopulations from noise and artifact in the NGS data since both would give rise to small VAFs (21).

**Figure 1.**
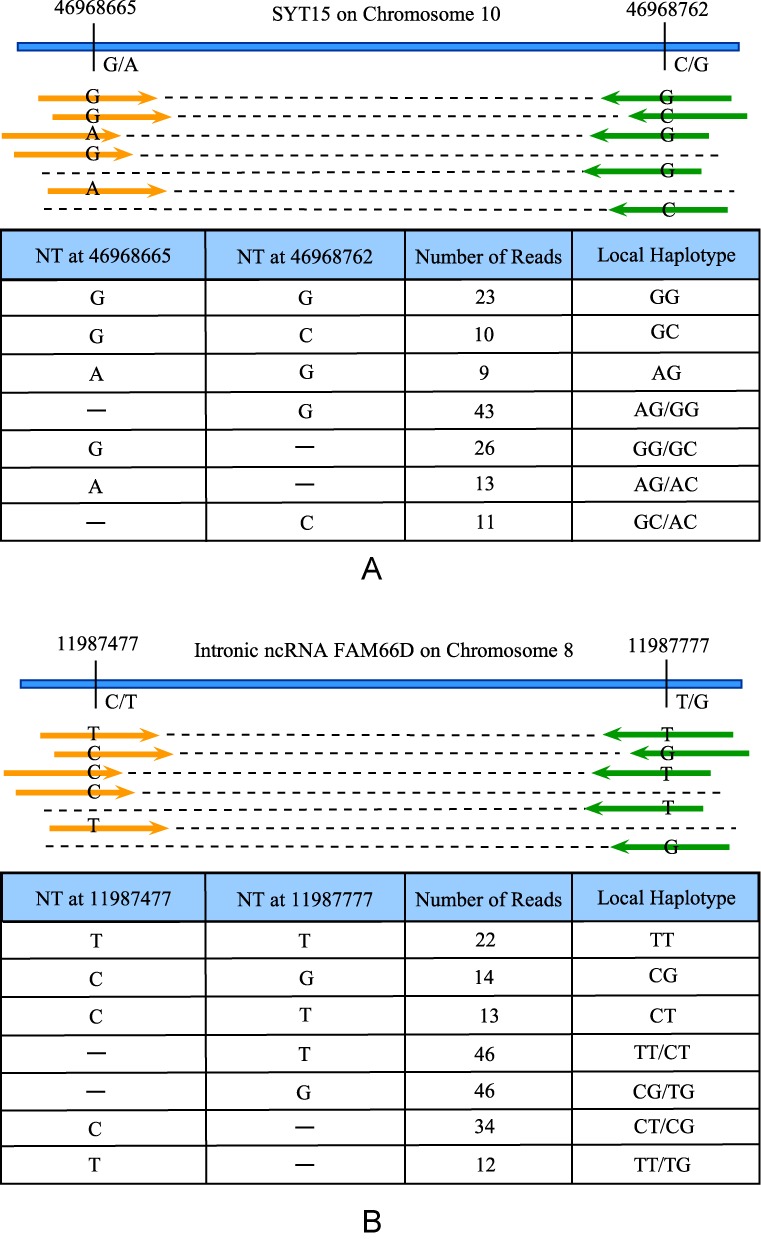
Two examples of LHVs based on direct observations of aligned short reads. The pairs of a single short read are marked with orange and green colored arrow, respectively. Panel (**A**): an LHV called from WES data of a normal blood sample. The haplotype consists of two SNVs separated by 97 bps in a coding region of gene *SYT15*. Among all the short reads mapped to this region, 23, 10 and 9 short reads are mapped to both SNVs and exhibit alleles GG, GC and AG, respectively. Due to the large count of the least frequent allele (AG) and the combined information from all other short reads, LocHap calls three local haplotypes (LH) with high statistical confidence, making it a variant (i.e., an LHV). Panel (**B**): an LHV called from WGS data of a normal blood sample from a normal individual (NA12878 in the CEU TRIO family in the 1000 genome project). The LH consists of two SNVs separated by 300 bps in an intronic region of an ncRNA FAM66D. Again, similar haplotype variants are seen based on the short reads mapped to both SNVs. In both examples, some reads are mapped to only one of the two SNVs. These reads provide partial information on the existence of certain haplotypes. For example, reads with ‘-G’ in panel (A) are only mapped to the second SNV with genotype ‘G’. They support that haplotypes AG or GG might be present in the sample. Hence, reads mapped to both SNVs and reads mapped to at least one SNV are used in the statistical models of LocHap.

We propose here a different approach. Instead of using SNVs, we consider ‘local haplotypes’ (LHs) for calling somatic mosaicism. An LH is a scaffold of multiple proximal SNVs (Figure 1). Examining paired-end DNA-sequencing data, we find that sometimes multiple SNVs are simultaneously mapped by the same short reads. The short reads provide linked genotypes for the SNVs. In Figure 1, two SNVs are considered in each example and some short reads cover both SNVs. Treating the scaffold of the two SNVs as an LH, shown in Figure 1, we observe three different genotypes with substantial read counts in each example. We call such an LH a local haplotype variant (LHV). The presence of LHVs across the genome is direct evidence supporting mosaicism and cellular heterogeneity because a homogeneous cell population can only manifest up to two haplotypes. Therefore, the key idea of examining an LH instead of an SNV allows for direct observation of more than two alleles in local genomes, a rare event for single loci but not for haplotypes. Based on this idea, we develop an open-source, ultrafast and powerful computational tool, ‘LocHap’, for identifying LHVs using deep DNA-sequencing data from a single biological sample. We construct rigorous statistics models that provide probability measure for the LHVs. We also introduce bioinformatic filters that account for the usual noise and artifact in NGS data. However, the noise and artifact are partially mitigated due to the use of LHVs instead of SNVs. We elaborate more on these points in the next section. LocHap can be applied to any DNA-sequence data using paired-end reads and only requires a binary alignment and mapping (*bam*) file, the associated index (*bai*) file and the corresponding variant call format (*vcf*) file (‘Materials and Methods’ section). These files are almost always generated from standard variant-calling pipelines. To facilitate downstream analyses and experimental validation, we introduce a new file format, the haplotype call format, or *hcf*, that contains a list of LHVs inferred by LocHap. An *hcf* file has a tab-delimited format similar to a *vcf* file, and can be viewed in popular visualization tools like integrated genome viewer (IGV) (22). The proposed *hcf* format is derived from the *vcf* format to facilitate visualization and interpretation. However, unlike *vcf* which contains SNVs and other genetic variants, *hcf* only contains information about LHVs, which is a scaffold of multiple local SNVs (each SNV is in the *vcf* file for the sample). Therefore, a non-empty *hcf* file presents information supporting genetically heterogeneous samples.

## MATERIALS AND METHODS

### Main idea

The basic idea of LHV calling is to probabilistically model short reads mapped to multiple proximal SNVs and look for multi-allelic loci. In other words, we search for proximal SNVs that are scaffolded by short reads and exhibit more than two alleles with high statistical confidence. For example, Figure 1A shows an LHV consisting of two SNVs, at chromosomal locations separated by only 97 bp. Examining data ‘horizontally’ across both SNVs, many reads scaffold the SNVs as they are mapped to both loci. There are three directly observed haplotypes, GG, GC, and AG, with read frequencies 23, 10, and 9, respectively. In addition, four other types of overlapping short reads cover only one of the two SNVs. Each type of short reads potentially supports the presence of one or two different haplotypes and collectively they provide information on how many and what haplotypes are present in the region. Using all the short reads, LocHap employs a Bayesian hierarchical model, performs statistical inference accounting for the noise in the data and filters dubious LHV calls based on false discovery rates (FDR) (23,24).

### Statistical methods

#### SNV segments

**Figure 2.**
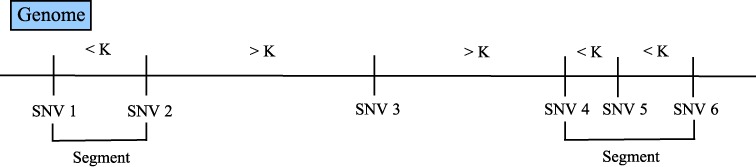
Illustration of DNA segments in LocHap. The first segment consists of two SNVs (SNV 1 and 2) and the second one has three SNVs (SNV 3, 4 and 5). SNV 3 is more than *K* base pairs from its adjacent SNVs 2 and 5, and therefore is not included in any segment.

LocHap uses DNA-Seq data and assumes that base calling, reads alignment and variant calling have been completed and *bam, bai* and *vcf* files are available for one or more samples. LocHap first constructs non-overlapping segments on the genome, each of which is a set of continuous base pairs (bps) and contains at least two proximal SNVs separated by no more than *K* bps apart. The segment is formed by starting at a SNV and extended to the next closest SNV as long as it is within *K* bps from the previous SNV. The segment ends if the next closest SNV is more than *K* bps away. Therefore, each segment starts and ends at a SNV, with potential multiple SNVs in between. A schematic illustration of DNA segmentation is shown in Figure 2.

Along the genome, we start with the first called SNV, and form as many segments as we can until we reach the last called SNV. LocHap allows any integer *K* set by users as the maximum distance between two adjacent SNVs. For short-read data, we allow *K* to vary between 50 and 1000. Changing *K* values will affect the size and number of segments. Usually the value of *K* can be set to reflect the insert length of the DNA sequencing experiment.

#### Probability model for LHV calling

LocHap analyzes each DNA segment separately. The goal of the analysis is to estimate the number and sequences of the haplotypes within the segment. Assume *N* numbers of short reads are mapped to the segment and each read overlaps with at least one SNV in the segment. Mapped reads that do not overlap with any SNVs are discarded since they do not contribute to the haplotype calling.

For a given segment, let *i* = 1, …, *N* be the index of the mapped short reads. Assume that *R* SNVs are present in the segment. We consider up to *L* = 2^*R*^ candidate haplotypes that can be formed by *R* SNVs. That is, we assume at each SNV, one can observe up to two different alleles (e.g., a reference and a variant allele). More than two alleles are rarely observed from short reads for an SNV and most of them are caused by sequencing error. We use *j* = 1, …, *K* to index possible haplotypes. The genotypes (nucleotide sequences) of each candidate haplotype are denoted by ${\boldsymbol h}_j = \lbrace h_{j1}, \ldots , h_{jR}\rbrace$, *j* = 1, …, *L*, where *h*_*jr*_ takes one of the four nucleotides, i.e., *h*_*jr*_ ∈ {*A, C, G, T*} for *r* = 1, …, *R*. For example, in Figure 1, *R* = 2 and *L* = 2^*R*^ = 4. In each example some short reads overlap with both SNVs. Specifically, for each short read *i*, use ${\boldsymbol s}_i = \lbrace s_{i1}, \ldots , s_{iR}\rbrace$ to denote an *R*-base DNA sequence of interest, where *s*_*ir*_ ∈ {*A, C, G, T, M*}; here *M* denotes a missing base readout when there is no overlap between a short read and an SNV. Let ${\boldsymbol s}= \lbrace {\boldsymbol s}_1, \ldots ,{\boldsymbol s}_N\rbrace$ be the set of all short reads. One could define an indicator *m*_*ir*_ = *I*(*s*_*ir*_ = *M*) to denote the missing base of ${\boldsymbol s}_i$ and set up a model *f*(*m*_*ir*_∣θ). We assume missing completely at random (MCAR) (25) , which leads to conditional independence in the posterior inference. That is, conditional on ${\boldsymbol s}$, parameters in the model describing target haplotypes are independent of ${\boldsymbol m}=\lbrace m_{ir}, \;\; i=1,\dots ,N, r=1, \dots , R\rbrace$, the vector of missing indicators. This greatly simplifies the inference procedure. The MCAR assumption is proper here since in NGS experiment, typically the missing base in *s*_*ir*_ is due to that read *i* is not aligned to base SNV *r*, which is caused by the limited read length as a technological limitation. Hence the missing mechanism in *s*_*ir*_ has nothing to do with what sequences are observed or not observed.

Using standard missing data notations, let:

\begin{equation*} {\boldsymbol s}^{{\rm obs}}=\lbrace s_{ir},\!\! \mbox{ where } m_{ir} =0 \mbox{ for } i=1,\ldots , N; \; r=1, \ldots , R\rbrace \end{equation*}

and

\begin{equation*} {\boldsymbol s}^{{\rm mis}}=\lbrace s_{ir},\!\! \mbox{ where } m_{ir} =1 \mbox{ for } i=1,\ldots , N; \; r=1, \ldots , R\rbrace \end{equation*}

denote the observed and missing DNA sequences for reads *i* at SNV *r*, respectively, for all *i*'s and *r*'s. Then $\lbrace {\boldsymbol s}^{{\rm obs}}, {\boldsymbol s}^{{\rm mis}}, {\boldsymbol m}\rbrace$ are the complete data, and $\lbrace {\boldsymbol s}^{{\rm obs}}, {\boldsymbol m}\rbrace$ are the observed data. We introduce a few additional notations needed for modeling. Denote {λ_*j*_ = 1} or {λ_*j*_ = 0} the event that haplotype *j* is present or absent in the sample, respectively. Apparently λ_*j*_'s are key parameters of interest. Intuitively, the sequence similarity between haplotype sequences ${\boldsymbol h}_j$ and short read sequences ${\boldsymbol s}_i$ provides information on which haplotype is present. For example, if ${\boldsymbol s}_i$ matches ${\boldsymbol h}_j$ in most of the *R* bases, it is likely ${\boldsymbol s}_i$ is generated from a DNA segment having haplotype *j*, thereby supporting the presence of the haplotype. To model the similarity, we denote $\mathcal {A}_j({\boldsymbol s}_i^{{\rm obs}})$ and $\mathcal {D}_{j}({\boldsymbol s}_i^{{\rm obs}})$ the set of agreeing and disagreeing bases between ${\boldsymbol s}_i$ and ${\boldsymbol h}_j$, respectively. Mathematically, they refer to:

\begin{eqnarray*} \mathcal {A}_j({\boldsymbol s}_i^{{\rm obs}}) = \lbrace r: s_{ir}=h_{jr}\rbrace ; \quad \mathcal {D}_j({\boldsymbol s}_i^{{\rm obs}}) = \lbrace r: s_{ir} \ne h_{jr} \; \& \; s_{ir} \ne M\rbrace. \end{eqnarray*}

Denote *I*() the indicator function and let

\begin{eqnarray*} \mathcal {B}_i = \lbrace r: s_{ir} =M\rbrace \mbox{ and } w_{i} = |\mathcal {B}_i| = \sum _{r=1}^R I(s_{ir} = M) \end{eqnarray*}

be the set of indices and number of missing bases of read *i*, respectively.

We propose a Bayesian probability model treating $({\boldsymbol s}^{{\rm obs}},{\boldsymbol m})$ as observed data and $\lbrace {\boldsymbol s}^{{\rm mis}}, \lambda _j\rbrace$ as unknown parameters. The inference is based on posterior probability that a haplotype *j* is present in the sample, $Pr(\lambda _j = 1 \mid {\boldsymbol s}^{{\rm obs}}, {\boldsymbol m})$. The higher value the probability takes, the more likely haplotype *j* is present. We will show next that this posterior probability can be calculated in a closed form.

Let ${\bf \lambda}=\lbrace \lambda _1, \ldots , \lambda _L\rbrace$ and ${\bf \lambda}_{-j}=\lbrace \lambda _1, \ldots , \lambda _{j-1}, \lambda _{j+1}, \ldots , \lambda _K\rbrace$ be the vector without the *j*-th component. The posterior probability $Pr(\lambda _j = 1 \mid {\boldsymbol s})$ can be calculated as follows:

(1) \begin{eqnarray*} Pr(\lambda _j = 1 \mid {\boldsymbol s}^{{\rm obs}}, {\boldsymbol m}) &=& Pr(\lambda _j = 1 \mid {\boldsymbol s}^{{\rm obs}}, \setminus\!\!\!{{\boldsymbol m}}) \propto \underbrace{p({\boldsymbol s}^{{\rm obs}} \mid \lambda _j=1)}_{\mbox{likelihood}}\, \underbrace{Pr(\lambda _j = 1)}_{\mbox{prior}} \nonumber \\ &=& \sum _{{\bf \lambda}_{-j} \in \mathcal {Y}_{L-1}} p({\boldsymbol s}^{{\rm obs}} \mid \lambda _j =1,{\bf \lambda}_{-j}) \, Pr(\lambda _j =1, {\bf \lambda}_{-j}) \nonumber \\ &=& \sum _{{\bf \lambda}_{-j} \in \mathcal {Y}_{L-1}} \left[\prod _{i=1} \underbrace{p({\boldsymbol s}_i^{{\rm obs}} \mid \lambda _j=1, {\bf \lambda}_{-j})}_{\mathcal {I}} \underbrace{Pr(\lambda _j = 1, {\bf \lambda}_{-j})}_{\mathcal {II}} \right], \nonumber \\ \end{eqnarray*}

where $\mathcal {Y}_{L-1}$ denotes the set of all binary (0 or 1) strings of length (*L* − 1). The first equation is due to the MCAR assumption. It can be shown (Supplementary Data) that:

\begin{eqnarray*} \mathcal {I} = \sum _{{j^\prime }=1}^L\left[ I(\lambda _{j^\prime }=1) \cdot c_{1i}(\lambda ) \frac{1}{\sum _{\tilde{j}=1}^L \lambda _{\tilde{j}}} \times \right. \nonumber \\ \left. \sum _{{\boldsymbol b}_i \in \lbrace A,C,G,T\rbrace ^{w_i}} \left\lbrace \prod _{r \in \mathcal {A}_{j^\prime }({\boldsymbol s}^{{\rm obs}}_i,{\boldsymbol s}^{{\rm mis}}_i={\boldsymbol b}_i)} (1-e_{ir}) \times \prod _{r \in \mathcal {D}_{j^\prime }({\boldsymbol s}^{{\rm obs}}_i,{\boldsymbol s}^{{\rm mis}}_i={\boldsymbol b}_i)} \frac{e_{ir}}{3} \right\rbrace \right], \end{eqnarray*}

where *e*_*ir*_ is the error probability for the DNA sequence called at base *r* on short read *i*. Typically *e*_*ir*_ is known from upstream analysis, e.g. in the form of *Phred quality score*. LocHap requires user-assigned values for *e*_*ir*_ with a default value of 0.001 (corresponding to a Phred score of 30). Alternatively, we recommend setting *e*_*ir*_ = 10^−*log(Ph)*^ where *Ph* is the Phred score at the base *r* of read *i* (26).

Next, the second term ($\mathcal {II}$) of Equation (1) is the product of independent prior term for each λ_*j*_ for all *j* = 1, ⋅⋅⋅, *L*,

\begin{eqnarray*} \lambda _j &\sim & \mbox{Beta-Bernoulli}(\alpha ,\beta ,n), \quad \mbox{ where } \lambda _j \in \lbrace 0,1\rbrace. \end{eqnarray*}

The Beta-Bernoulli prior for λ is the marginal density of hierarchical construction in which

\begin{equation*} \lambda \mid \tau \sim \mbox{Bernoulli}(1, p); \qquad \tau \sim \mbox{Beta}(\alpha ,\beta ). \end{equation*}

Integrating out τ, we get a Beta-Bernoulli prior given by

(2) \begin{equation*} Pr(\lambda = 1) = \frac{\Gamma (1+\alpha )\Gamma (\beta )}{\Gamma (1+\alpha +\beta )}\, \frac{\Gamma (\alpha +\beta )}{\Gamma (\alpha )\Gamma (\beta )}. \end{equation*}

To reflect the weak prior belief that a random haplotype has a low prior probability to be present in a sample, we set α = 0.05 and β = 1 so that *a priori* the probability that haplotype *j* is present is only 5%.

#### FDR-based inference and calibration of *e*_*ir*_

Denoting $\xi _j = Pr(\lambda _j=1 \mid {\boldsymbol s}^{{\rm obs}}, {\boldsymbol m})$ the posterior probability that haplotype *j* is present in the sample. Posterior inference is based on selecting the haplotypes with the largest ξ_*j*_ subject to an FDR threshold. For example, with a desired FDR threshold of *f*_0_, compute

(3) \begin{equation*} j*=\max \left\lbrace j: \frac{\sum _{k < j} (1-\xi _{(k)})}{|\lbrace k: k<j\rbrace |} < f_0\right\rbrace \end{equation*}

where ξ_(*k*)_ is the ordered statistics with decreasing order and |{*set*}| is the cardinality of the set. Then select all the haplotypes with ξ_*j*_ > ξ_j*_. Such a selection procedure is optimal (23,24) in controlling posterior expected FDR.

All the parameters in the proposed Bayesian model are estimated directly. The models only depend on one calibration parameter, *e*_*ir*_, which must be given. The error rate *e*_*ir*_ captures the quality and Phred quality score from base calling, an upstream analysis. In most cases, a Phred quality score of >30 is considered of high quality for a base, which translates to *e*_*ir*_ < 0.001 by definition (http://en.wikipedia.org/wiki/Phred_quality_score). Also, shown in Ji *et* *al*. (26) a higher error rate leads to more noisy inference, in our case, less confidence on haplotype calls. As an example, Supplementary Data Table S1 provides a simulated dataset in which each row represents a short read and its called bases and a ‘−’ sign represents a missing base. Applying our proposed model with *e*_*ir*_ = 0.001 for all reads and bases, we infer that three LHs, *AA, GA* and *GG*, are present in the sample using an FDR threshold *f*_0_ = 0.01. If we increase the *e*_*ir*_ to 0.2 we obtain only one LH *AA* with *f*_0_ = 0.01. If we use *e*_*ir*_ = 0.14, we get two significant LHs *AA* and *GG*.

In LocHap, we remove reads having a mapping quality score <30; we also consider a base missing if the Phred quality score of base calling is <30. These two steps ensure the high quality of the reads and bases used in the statistical inference. Then we take a conservative value of 0.001 for all the *e*_*ir*_'s as the default setting. This is a conservative choice since 0.001 is the largest possible *e*_*ir*_ value after the above read filtering. As a less conservative choice, one could use the provided *e*_*ir*_ for each base and read from the *bam* file.

### Efficient computational algorithm

Posterior inference of LHVs centers at the calculation of $Pr(\lambda _j= 1 \mid {\boldsymbol s}^{{\rm obs}})$. We re-list Equation (1) again to facilitate the subsequent discussion, given by

(4) \begin{eqnarray*} Pr(\lambda _j = 1 \mid {\boldsymbol s}^{{\rm obs}}) \propto \sum _{{\bf \lambda}_{-j} \in \mathcal {Y}_{L-1}} \left[ \underbrace{\prod _{i=1}^{N} \underbrace{Pr({\boldsymbol s}_i^{{\rm obs}} \mid \lambda _j=1, {\bf \lambda}_{-j})}_{\mathcal {I}} \underbrace{Pr(\lambda _j = 1, {\bf \lambda}_{-j})}_{\mathcal {II}}}_{\mathcal {III}} \right] \end{eqnarray*}

As mentioned before, if the number of SNVs is *R*, then the number of possible haplotypes *L* = 2^*R*^, assuming up to two alleles can be observed at each SNV. Correspondingly, we have *L* number of λ_*j*_'s to estimate and the total different configurations of all the λ_*j*_'s is $2^L = 2^{2^R},$ a super exponent of *R*. Therefore, when *R* is slightly increased, say from 2 to 4, the number of configurations to be calculated increases from 64 to 65, 536. This super-exponential increment calls for efficient computation.

A straightforward way to calculate the right hand side of the Equation (4) would follow the derivation in the previous section, resulting in computing multiple loops of summations and products. It would be time consuming. We take a more efficient approach. For each *j* = 1, 2, …, *L*, summing over all the binary configurations of ${{\bf \lambda}}_{-j}$ amounts to 2^*L* − 1^ many sums. Each term under the outer sum is denoted by $\mathcal {III}$ in (4). A straightforward computation of (4) would calculate term $\mathcal {III}$ (L*2^*L* − 1^) times for all the *j*. Same amount of computation is also required for calculation of $Pr(\lambda _j=0 \mid {\boldsymbol s}^{{\rm obs}})$ for all *j* = 1, 2, …, *L*. But careful examination of the terms to be added reveals that some terms are repeatedly calculated *L* times. For example, assume *L* = 4. In calculating the probability for the event $Pr(\lambda _1=1 \mid {\boldsymbol s}^{{\rm obs}})$, we have to sum over all the other 2^*L* − 1^ = 8 configurations of ${{\bf \lambda}}_{-1}$. Let us take one specific configuration from that set of eight configurations, ${{\bf \lambda}}_{-1} = 101$ (meaning the three elements in ${\bf \lambda}_{-1}$ take values 1, 0 and 1, respectively). When λ_1_ = 1, the full vector ${\bf \lambda}$ takes 1101. However, the value 1101 will also show up in the computation of $Pr(\lambda _2=1 \mid {\boldsymbol s}^{{\rm obs}})$ with ${{\bf \lambda}}_{-2} = 101$, $Pr(\lambda _3=0 \mid {\boldsymbol s}^{{\rm obs}})$ with ${{\bf \lambda}}_{-3} = 111$ and $Pr(\lambda _4=1 \mid {\boldsymbol s}^{{\rm obs}})$ with ${{\bf \lambda}}_{-4} = 110$. Therefore, we only need to compute the joint probability of ${\bf \lambda}=\lbrace 1101\rbrace$ once and re-use it for the other three terms. Similarly, for all other possible configurations of ${\bf \lambda}$, we only need to compute it once. The straightforward way of computation would calculate each configuration four times.

Once all 2^*L*^ configurations are calculated, we add up the probabilities from appropriate configurations in order to calculate the probability $Pr(\lambda _j=1 \mid {\boldsymbol s}^{{\rm obs}})$. We first put decimal indices against all the configurations of ${\bf \lambda}$ from 0 to (2^*L*^ − 1) by treating the first position as the most significant bit of a binary string and convert the binary string to its decimal equivalent number. For example, the decimal index of ${\bf \lambda}= \lbrace \lambda _1=1, \lambda _2=1, \lambda _3=0, \lambda _4=1\rbrace$ is 13. Denote each configuration by *C*_*l*_ where *l* = 0, 1, …2^*L*^ − 1. Once indexing is done, then for each event we sum up the probabilities for a fixed (computed beforehand) set of indices of configurations. For example, for the computation of $Pr(\lambda _2=1 \mid {\boldsymbol s}^{{\rm obs}})$ the set of indices is {4, 5, 6, 7, 12, 13, 14, 15}. Similarly for $Pr(\lambda _3=0 \mid {\boldsymbol s}^{{\rm obs}})$ that set is {0, 1, 4, 5, 8, 9, 12, 13}. Denote the set of indices for computing $Pr(\lambda _1=1 \mid {\boldsymbol s}^{{\rm obs}} )$ and $Pr(\lambda _1=0 \mid {\boldsymbol s}^{{\rm obs}} )$ by $\mathcal {V}_{j1}$ and $\mathcal {V}_{j0}$, respectively. Below, we propose Algorithm 1 for computing (4).


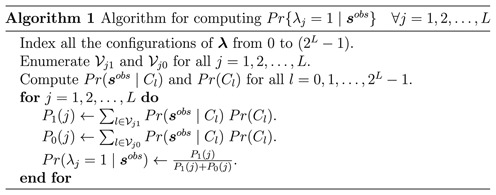


Calculation of $Pr({\boldsymbol s}^{{\rm obs}} \mid C_l)$ for all *l* = 0, 1, …, 2^*L*^ − 1 in Algorithm 1 is carried out with an additional algorithm that takes advantage of the structured probability formulation. The detail is shown in Supplementary Data. Calculation of *Pr*(*C*_*l*_) is trivial based on the independent prior of λ_*j*_'s. Because of the closed-form derivation for (4) and efficient computation algorithms, LocHap is ultra-fast in analyzing whole-genome and whole-exome data, taking usually less than a minute for a whole genome.

### LocHap pipeline

**Figure 3.**
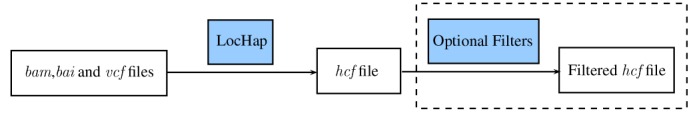
Overview of the LocHap pipeline.

A computational pipeline (Figure 3) supports LocHap applications. LocHap analyzes one sample at a time and can be used sequentially or in parallel for the analysis of multiple samples. For a single sample, the input of LocHap includes (i) a *bam* file with the associated index *bai* file and (ii) a corresponding *vcf* file that contains the SNVs in the sample, called by any standard variant calling algorithm, such as *GATK's* (27–29) UnifiedGenotyper tool. The output of LocHap is a set of LHVs stored in *hcf* files, one for each sample.

#### Haplotype call format (hcf)

Each line in the *hcf* file contains information about one particular LHV segment. Below is a line in an *hcf* file from analysis of real-world data.


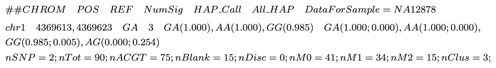


Same as *vcf*, an *hcf* file is a tab-delimited text file. After the initial header fields, each line in the hcf file represents a LH (might not be a variant) and has seven column fields. Also, at the end of each *hcf* file, a summary stating the total number of SNVs in the *vcf* file, number of segments with zero significant haplotypes, one significant haplotype, two significant haplotypes and so on, are given (see Supplemental Data).

### Post processing

The inferred LHVs can be filtered to remove false positives caused by artifact and noise in the sequencing data. The filters are devised to remove dubious reads and SNVs. In NGS data, known artifact and error affect SNV calling (21) by erroneously calling or aligning the bases on short reads. However, they do not artificially create additional LHs which require more sophisticated changes to the bases of short reads across multiple SNVs. The typical artifact and error often changes the base calling or alignment for a single locus and usually affect all the short reads in the region. Therefore, LHV-based inference is less prone to the errors and artifacts for SNV calling. Nonetheless, we apply a set of optional and customizable filters (Supplementary Data) with different stringency levels for post-processing of the LHVs in the output *hcf* files. Currently, despite the large amount of effort directed by the community (21) the noise and error in NGS experiments and data preprocessing cannot be statistically modeled or quantified. There is no consensus on filtering the variant calls from various analysis pipelines. We present a conservative filtering pipeline that is heavily biased toward reducing FDR, so that reported LHVs are of high confidence. The proposed filtering depends on various parameters that can be modified to enforce different degrees of filter stringency. A more stringent filter results in fewer LHVs at the end.

The proposed filters can remove SNVs that are too close to each other (within, say 50 bps) and SNVs that are close to other types of variants such as indels. It has been noted (30,31) that these variant calls are not trustworthy due to artifacts and base calling errors in the data. In addition, our filters can remove SNVs for which most reads are aligned to the SNV at a base near the end of the reads. The reason is that bases called toward the end of a read are usually of low quality, which then affect the reliability of the alignment. Lastly, SNVs mapped by reads with strand bias (32) are also filtered.

### Integrated genome viewer (IGV) compatibility

**Figure 4.**
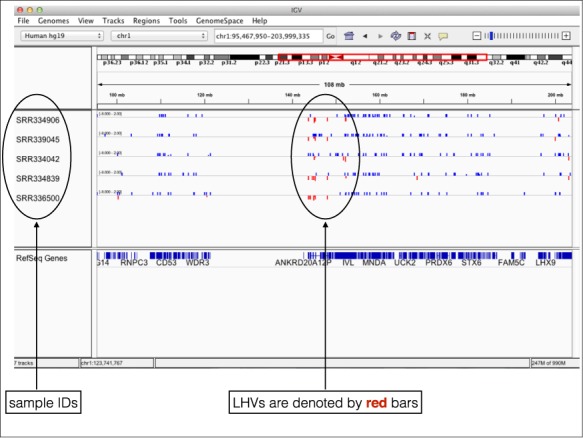
Visualization of *hcf* files in IGV.

For better visualization, we provide an additional IGV-compatible format so that LHV segments can be visually examined in the popular genome visualization tool IGV (22). A snapshot of five *hcf* files in IGV is shown in Figure 4. The details of the corresponding command is given in *Quick Manual* (http://compgenome.org/lochap/code_release/QuickManual-LocHap-release-v1.0.pdf). Note that the LHVs are shown by *red* bars and non variants are shown by *blue* bars.

## RESULTS AND DISCUSSION

### Simulation

We first demonstrate the utility of the proposed statistical model using simulated data. In all of the following examples we used *e*_*ir*_ = 0.001 as the default value and FDR threshold was set at *f*_0_ = 0.01. Also we assumed that the probability of observing more than two different alleles at a particular locus in a genome was considered rare. This is assumed because of the small chance of having a point mutation occurring twice at the same nucleotide (33–35). All of the simulation examples were based on short reads data generated for a single segment. Also, we only show examples with a small number of short reads. When a large number of reads were simulated, the proposed models performed very well, easily recovering the simulation truth. Tabulated posterior probabilities for all the scenarios are provided in the Supplementary Tables S1–S9.

#### Simulation Scenario 1

We generated eight short reads covering two SNVs. Assuming at each SNV that only two alleles could be observed, there were 2^2^ = 4 possible haplotypes. The simulated short reads had genotypes

\begin{equation*} D = \lbrace GA, GA, GA, GA, GC, GC,AC,AC\rbrace. \end{equation*}

Applying LocHap, we inferred three significant haplotypes with the following sequences and posterior probabilities.

\begin{equation*} \lbrace GA: 1.00, \; GC: \;>0.99, \; AC: \; >0.99 \rbrace \end{equation*}

#### Simulation Scenario 2

In this scenario, we generated eight short reads, each of which only covered one of the two SNVs.

\begin{equation*} D = \lbrace A-, -A, G-, G-, -G, -G, -G,-G\rbrace. \end{equation*}

Here the read labeled ‘*A* −’ indicated that the first SNV position had a readout *A* and the read did not cover the second SNV position. Hence, we used ‘−’ sign to represent a missing genotype. Using LocHap, no haplotypes can be inferred to be present based on the FDR threshold *f*_0_ = 0.01.

#### Simulation Scenario 3

In this scenario, we simulated five short reads covering three SNVs with genotypes given by

\begin{equation*} D = \lbrace AGA, AGA, AGC, AGC, GAC\rbrace. \end{equation*}

LocHap called three significant haplotypes {*AGA, AGC, GAC*} with posterior probabilities all >0.99.

#### Simulation Scenario 4

In this scenario, we generated eight short reads covering three SNVs, given by

\begin{equation*} D =\lbrace AAA, AAT, ACA, ACT, GAA, GAT, GCA, GCT\rbrace. \end{equation*}

LocHap did not identify any significant haplotypes. This is due to the lack of strong evidence for any of the haplotypes as each of them is supported by only one read. The proposed model correctly recognized the uncertainty in the data and did not provide statistical significance for any haplotypes.

#### Simulation Scenario 5

This scenario is similar to scenario 4 but here we generated five short reads each for the haplotypes AAA, AAT and ACA. The data is given by,

\begin{eqnarray*} &&D = \nonumber \\ &&\lbrace AAA {\times} 5, AAT {\times} 5, ACA {\times} 5, ACT, GAA, GAT, GCA, GCT\rbrace. \end{eqnarray*}

Although LocHap did not find any significant haplotypes in the previous scenario, LocHap called three significant haplotypes {*AAA, AAT, ACA*} with posterior probabilities all equal to 1.00 because of more reads are generated for these three haplotypes. It is easy to see that with more number of reads our inference model would work more accurately.

#### Simulation Scenario 6

This scenario is similar to scenario 1 but here we generated five times more number of short reads for every categories. The data is given by,

\begin{equation*} D = \lbrace GA {\times} 20, GC {\times} 10, AC {\times} 10\rbrace. \end{equation*}

Applying LocHap, we again inferred three significant haplotypes with posterior probabilities all equal to 1.00.

#### Simulation Scenario 7

This scenario is same as the real-life data in Figure 1A. The data is given by

\begin{eqnarray*} &&D = \nonumber \\ &&\lbrace GG {\times} 23, GC {\times} 10, AG {\times} 9, -G {\times} 43, G- {\times} 26, A- {\times} 13, -C {\times} 11\rbrace. \end{eqnarray*}

Applying LocHap, we inferred three significant haplotypes {*GG, GC, AG*}with posterior probabilities all equal to1.00.

#### Simulation Scenario 8

This scenario is same as the real-life data Figure 1B. The data is given by,

\begin{eqnarray*} &&D = \nonumber \\ &&\lbrace TT {\times} 22, CG {\times} 14, CT {\times} 13, -T {\times} 46, -G {\times} 46, C- {\times} 34, T- {\times} 12\rbrace. \end{eqnarray*}

Applying LocHap, we again inferred three significant haplotypes {*TT, CG, CT*} with posterior probabilities all equal to 1.00.

All eight scenarios show that LocHap performs well. When the number of reads increases, the confidence in the statistical inference also increases.

### Three DNA-seq datasets

We applied LocHap to three different datasets, among which two were public and one from our own in-house validation experiments. We provide main findings next and put analysis details in the Supplementary Data.

#### Head and neck cancer (HNC) data

We analyzed whole exome sequencing (WES) data of 30 matched tumor and blood sample pairs (total 60 samples) from patients with head and neck cancer (HNC) (36). Whole exome Sequence Read Archive (SRA) files of matched tumor and normal samples were downloaded from the SRA (http://www.ncbi.nlm.nih.gov/sra). Standard bioinformatics analyses were performed to extract *fast-q* sequences, map short reads and call SNVs. We generated *bam* files (one per sample) and a *vcf* file for all the samples. The *bam* files contained short read sequences and alignments and the *vcf* file contained SNV calls of all the samples. The *bam* files with associated *bai* and *vcf* files were provided to the LocHap pipeline, which subsequently generated 60 *hcf* files, one for each sample.

**Figure 5.**
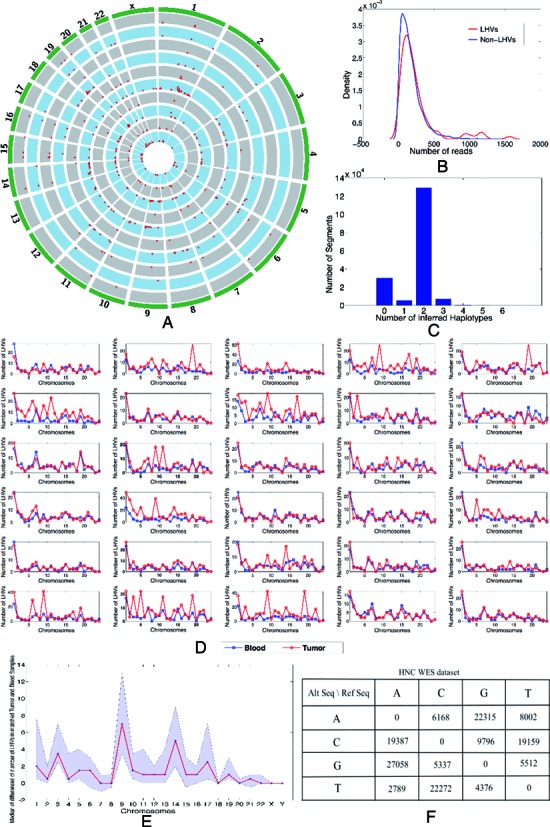
LHV calling for a head and neck cancer (HNC) WES dataset with 30 pairs of matched tumor and normal samples. (**A**) A circos plot of prevalence of LHVs for five arbitrarily selected sample pairs. Each red dot indicates the existence of at least one LHV in the corresponding exonic region of 1M bps. The height of a red dot indicates the number of LHVs present in the segment of 1M bps long. A pair of matched tumor and normal samples are arranged as adjacent circles with grey and blue color, respectively. (**B**) Comparison of read depth for genome regions with and without LHVs. No apparent difference is observed. (**C**) Histogram showing the frequencies of DNA segments (vertical axis) with different numbers of haplotype calls (horizontal axis). Most regions have up to two haplotypes, i.e., no variants. Regions with greater than two haplotypes are variants implying genome mosaicism. (**D**) A total of 30 line plots, one for each pair of matched tumor (red) and normal (blue) samples from an individual patient. The number of LHVs is shown for each chromosome for each patient. In general, tumors exhibit more LHVs implying more mosaicism. (**E**) Summary of (**D**). For each chromosome, a blue dot is the median of the difference in the number of LHVs between tumor and its matched normal sample across 30 patients; point-wise confidence intervals are shown as purple bands. Tumors show much higher frequencies of LHVs on chromosomes 9, 14 and 17, indicating potential disease-related variations on these regions. (**F**) Summary of sequence mutations for the SNVs within called LHVs. Transitions are much more prevalent than transversions.

**Figure 6.**
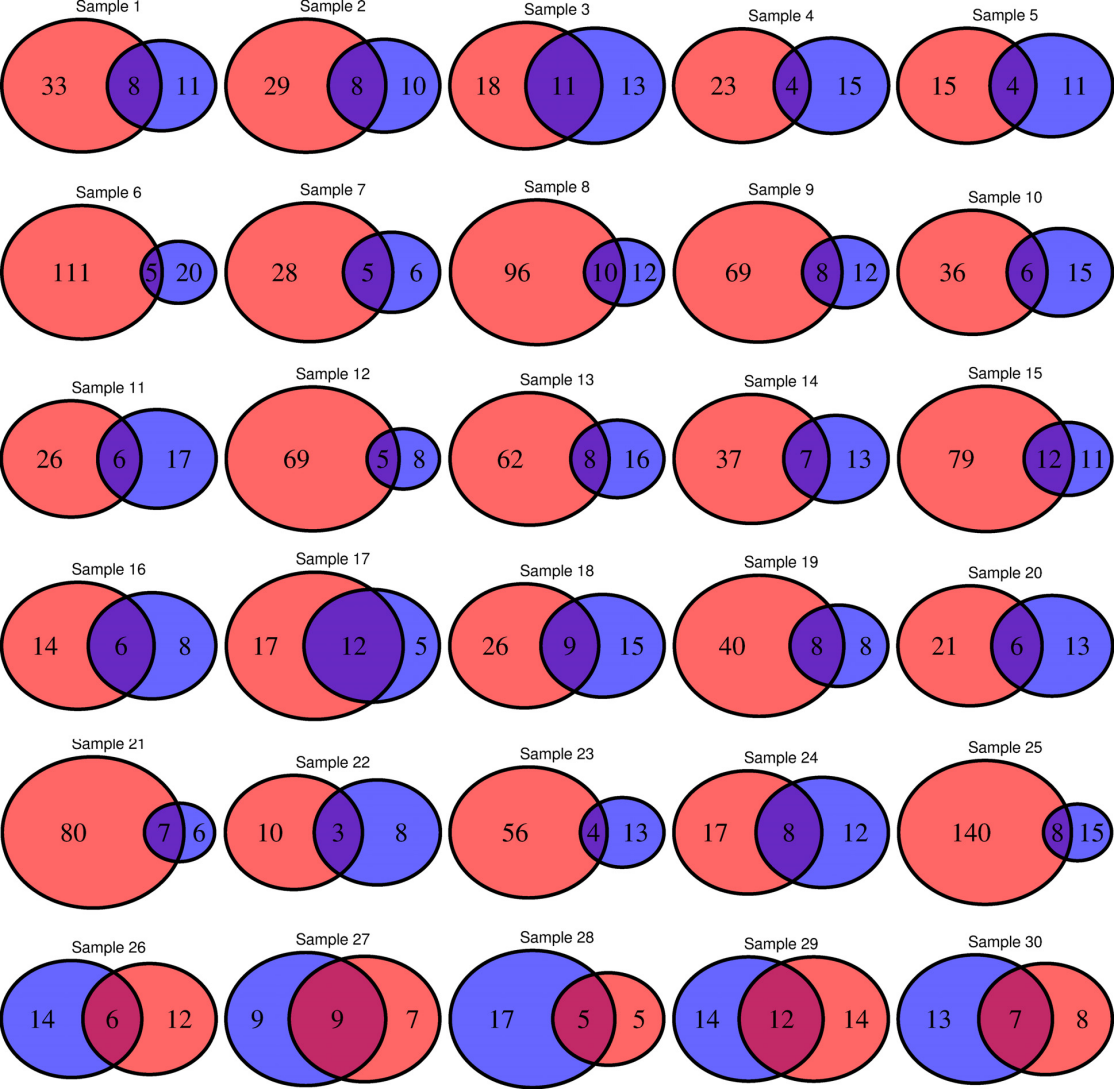
Venn diagrams showing the overlap of LHV calling for a HNC WES dataset with 30 pairs of matched tumor and normal samples. For each pair, LHV counts for tumor and the matched normal sample are shown in red and blue color, respectively. In most of the samples, number of LHVs in tumor is greater than that of the matched normal except for the last five samples where the numbers are comparable or number of LHVs found in the tumor sample is less the corresponding number in the matched normal sample.

Figure 5A shows a circos plot (37) of the called LHVs. Most LHVs are located in different genomic regions across patients, suggesting somatic mutations occurred randomly across the genome. Also, the fact that called LHVs are mostly different between patients indirectly shows that LHV calling is not driven by artifact and noise in the NGS data. The reported LHVs all passed the aforementioned noise filtering with stringent criteria. Read depth of one exome of a normal sample is shown in Figure 5B. A few LHVs are mapped with large numbers of reads but overall the read depths between LHVs and non-variant regions are comparable. Most LHs are not LHVs, having no more than two genotypes and most LHVs possess three genotypes (Figure 5C). Tumors in general possess more LHVs than corresponding normal samples (Figure 5D) and chromosomes 9, 14 and 17 are ‘hotspots’ for LHVs exhibiting higher frequencies in tumors than blood samples (Figure 5E). Transitions are more frequent than transversions (Figure 5F), as expected. Finally, overlapping LHVs are present in both tumor and the matched blood samples for each of the 30 patients (Figure 6), while the tumor and blood samples also possess unique LHVs of their own.

Table 1 summarizes the statistics from the unfiltered *hcf* file from one particular normal blood sample.

**Table 1.** Statistics from *hcf* file of a sample from HNC dataset

No. of variants	Segments with no. of significant haplotypes									Segments with more than three variants
(lr)2–10	0	1	2	3	4	5	6	7	8	(Not analyzed)
137886	460	85	2070	90	11	0	0	0	0	457

#### CEU-TRIO data from 1000-genomes project

**Figure 7.**
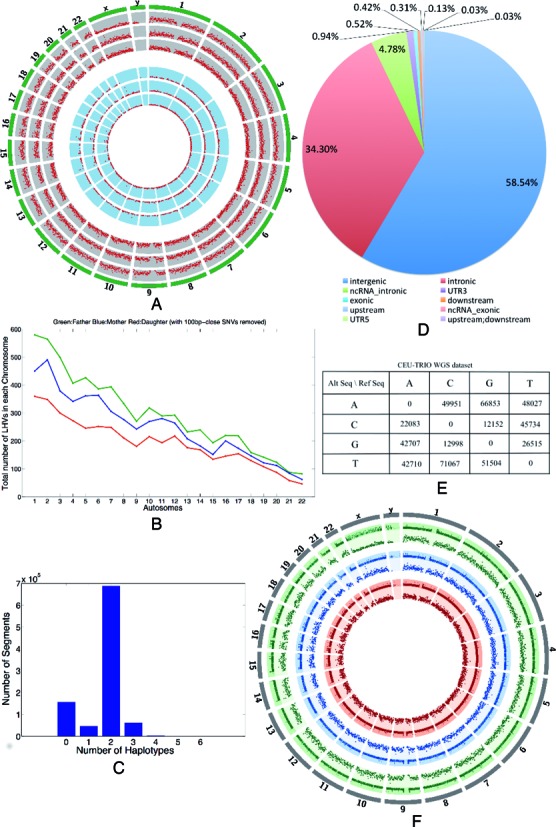
LHV calls for normal samples from a CEU trio of father, mother and daughter in the 1000 genome project based on WGS data. (**A**) A circos plot of prevalence of LHVs. Outer three arcs and inner three arcs represent results of TRIO samples filtered by type III filter and type I filter, respectively. See ‘Materials and Methods’ section for details of the filters. Each red dot indicates the existence of at least one LHV in the corresponding genomic region of 1M bp. The height of a red dot indicates the number of LHVs present in the region. (**B**) Comparison of the three family members in the number of LHVs per chromosome. The daughter has the smallest and the father has the largest number of LHVs in all chromosomes (autosome). (**C**) Histogram showing the frequencies of DNA segments (vertical axis) with different numbers of haplotype calls (horizontal axis). Most segments have up to two haplotypes indicating no variant. Segments with greater than two haplotypes are variants implying genome mosaicism. (**D**) Functional annotations of the genome regions where LHVs are found. Most are intergenic and intronic, with <1% LHVs in exons. (**E**) Summary of sequence mutations for the SNVs within called LHVs. Transitions are much more prevalent than transversions. (**F**) Copy number calls based on CNVnator (38) are directly compared with LHVs for all three family members. In most cases, there are no copy number variations on genome regions where LHVs are found. Copy numbers are represented in the outer arc and LHVs are shown in the adjacent inner arc in the same color for each sample.

We applied LocHap to whole genome sequencing (WGS) data of a CEU TRIO family of father, mother and child from the 1000-Genomes project (http://www.1000genomes.org/). The analysis procedure was identical to the HNC data, except here we have WGS data from three members of a family. Genome-wide LHVs (Figure 7A) are found in all three individuals with father having the largest number of LHVs and daughter the smallest (Figure 7B). This reflects the evolutionary conjecture that somatic mutations emerge over time as a result of accumulating mitotic errors and that the longer an individual lives, the more likely somatic mosaicism is seen on the genome (18). Similar to the results obtained in the previous analysis of cancer WES data, most LHs are not LHVs and most LHVs possess three genotypes (Figure 7C). Most LHVs reside in intergenic and intronic regions with less than 1% in exons (Figure 7D). Here again transitions are more prevalent than transversions (Figure 7E). We called copy number variations (CNVs) using CNVnator (38). Convincingly, CNVs are not observed for most LHVs regions, suggesting that the LHVs are not associated with CNVs, a potential confounder for LHV calling. There are almost no overlapping LHVs across the three family members. This is expected since LHVs are results of somatic mutations, which do not usually re-occur in different individuals. Under the most stringent filter, on average 400–500 LHVs are reported per genome using the WGS data in CEU trio compared with 4–5 per exome using the WES data from the HNC sample.

Table 2 summarizes the statistics from the unfiltered *hcf* file from one particular sample (NA12891) in this dataset.

**Table 2.** Statistics from *hcf* file of a sample from CEU trio dataset

No. of variants	Segments with no. of significant haplotypes									Segments with more than three variants
(lr)2–10	0	1	2	3	4	5	6	7	8	(Not analyzed)
6378548	43322	17216	232750	22839	1430	54	2	0	0	196078

#### Validation

In order to validate our results, we sequenced whole blood DNA from three members of a parent–child trio using two different sequencing platforms, Complete Genomics, Inc. (CGI) (http://www.completegenomics.com/documents/DataFileFormats_Standard_Pipeline_2.5.pdf, http://cgatools.sourceforge.net/docs/1.8.0/cgatools-user-guide.pdf) and Illumina whole genome sequencing (ILMN) (http://www.illumina.com/applications/sequencing.html). All members of the trio were healthy. Their blood samples were collected between 2007 and 2012 and sequenced by CGI in 2012 (39). We also sequenced DNA from the same three samples using the ILMN platform in 2014. Because ILMN and CGI utilized different sequencing technologies and the sequencing experiments were performed at separate times by more than 2 years apart, results from the two sequencing experiments serve to validate each other.

**Figure 8.**
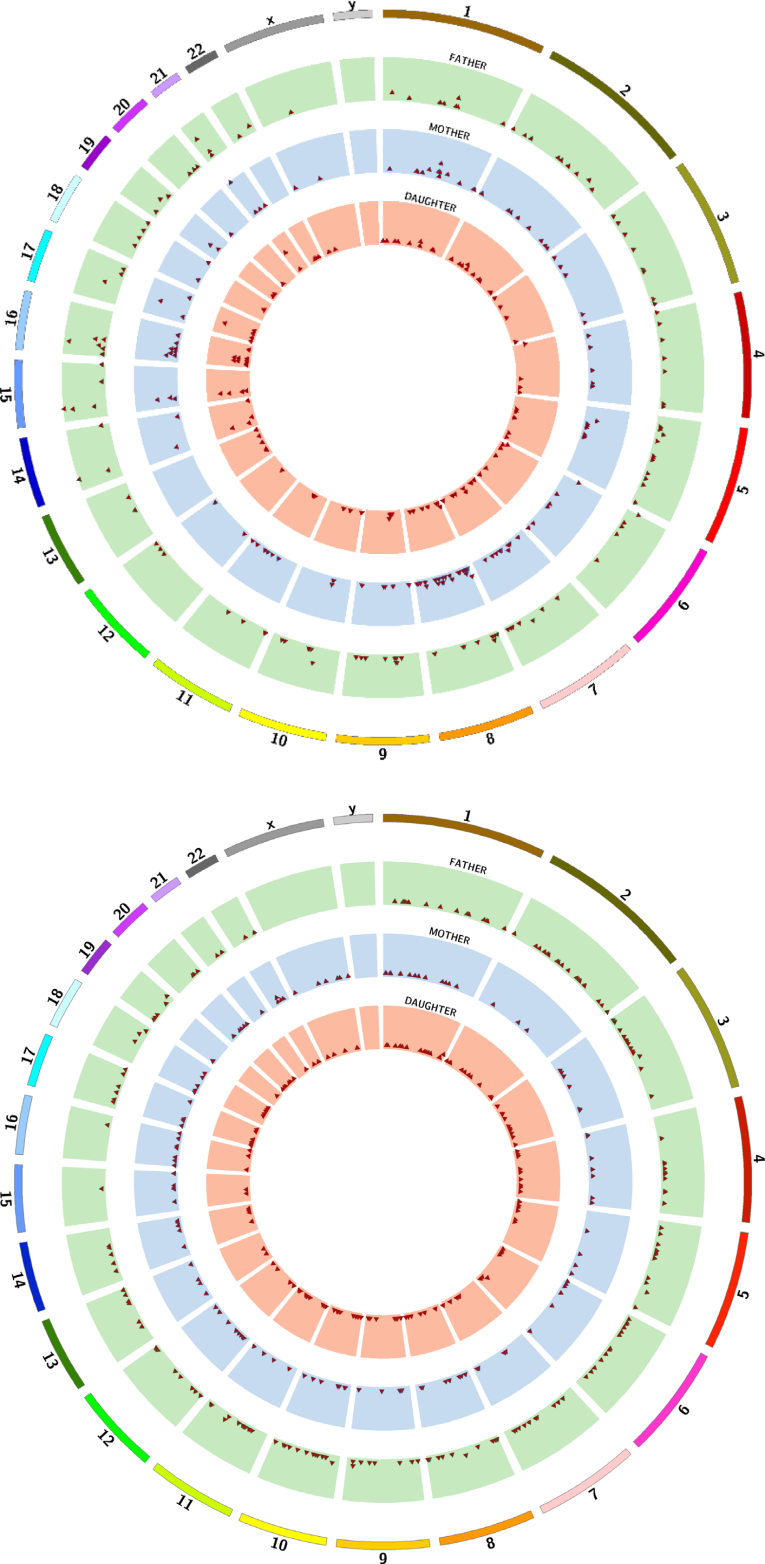
Summary of the LHVs found in the father, mother and daughter of a family based on both Illumina (ILMN) and CGI data. The ages are 57, 47 and 22, respectively. Top panel: A circos plot of prevalence of LHVs for ILMN data. The three colored rings describe the genome-wide prevalence and locations of the LHVs for the three family members. Each red triangle dot indicates the existence of at least one LHV in the corresponding genomic region of 1M bp. The higher a red dot resides, the larger number of LHVs present in the region. Bottom panel: a circos plot of prevalence of LHVs for CGI data. The plot follows the same arrangement as in the top panel.

**Figure 9.**
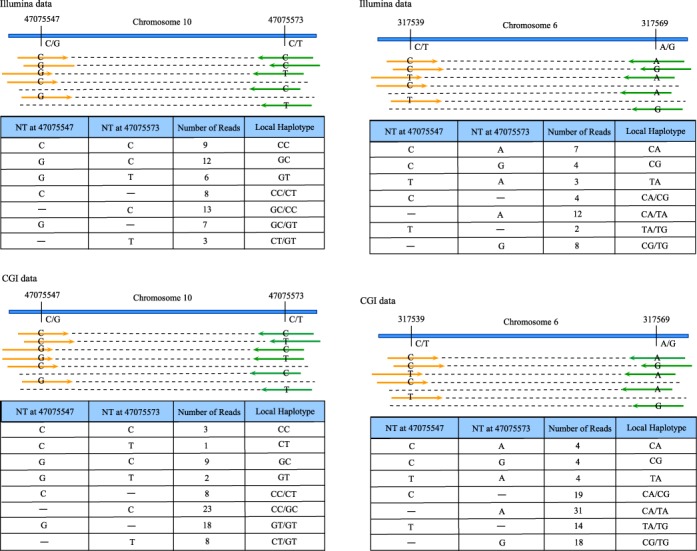
Two examples of LHVs that overlap between CGI and ILMN data. LHV1 consists of two single nucleotide variants (SNVs) separated by 26 bps on Chromosome 10. For the ILMN data (top tables), among all the short reads mapped to this region, 9, 12 and 6 short reads are mapped to both SNVs and exhibit genotypes CC, GC and GT, respectively. For the CGI data (bottom tables), those numbers are 3, 9 and 2 respectively. Also, many other reads are mapped to one of the SNVs for both data, which reinforces the finding. Statistical inference shows high significance supporting more than two haplotypes in the region. LHV 2 consists of two SNVs separated by 30 bp. For the ILMN data, 7, 4 and 3 reads are mapped to both SNVs with genotypes CA, CG and TA, respectively. Those numbers are 4, 4 and 4 for the CGI data.

NGS data produced by both technologies were analyzed using the LocHap pipeline. At the end, for each of the two datasets we generated a list of LHV. We then overlapped the two lists of LHVs, and identified shared LHVs between both datasets. Nine LHVs overlapped between the two datasets in the child; 10 LHVs overlapped in the mother and 15 LHVs overlapped in the father. We applied highly stringent filtering rules (Supplemental Data) to ensure high quality of the reported LHVs, although such filtering could also remove true LHVs with weak confidence. Also, many LHVs were excluded due to insufficient evidence from the CGI data. Figure 8 shows the locations of LHVs for the CGI and ILMN data. Figure 9 presents two LHV examples that are shared between CGI and ILMN data. For these two LHVs, the short reads provide direct evidence of somatic mosaicism—the reads suggest that at least three LHs must be present.

These analyses provide evidence supporting our hypothesis that normal cells in a healthy person could be genetically heterogeneous and possess distinct populations of somatic cells, a phenomenon also observed in (5). Specifically, in all three individuals there are LHVs that are discovered by independent sequencing platforms from different experimentalists at different times.

### DISCUSSION

Through a novel means of analyzing NGS data, LocHap attempts to reveal potential somatic mosaicism in the form of LHVs. We implement Bayesian hierarchical models that borrow strength from the mapped short reads to infer the number and sequences of LHVs genome-wide. In applications of LocHap using deep-sequencing data, we provide evidence that supports the existence of normal somatic mosaicism (NSM) and tumor somatic mosaicism (TSM) at single-nucleotide level. Applying LocHap to 30 matched blood and tumor samples, we find LHVs in exomes of normal blood and tumor samples. The frequencies of LHVs are in general higher in tumor samples (one-sided paired *t*-test, *P*-value < 0.0001). Performing the analysis on CEU trio from the 1000-genome project, we confirm the findings of genome-wide LHVs and also identify an increasing trend of LHV occurrences with aging (chi-squared test (40) for trend, *P*-value < 0.0001). Based on our results, we propose three hypotheses that deserve future investigation.

- Similar to cancer cells, non-cancer cells undergo random mutation events that could potentially lead to subclonal cell populations, resulting in genome-wide somatic mosaicism within individuals.
- The probability of acquiring NSM increases with the age of an individual, owning to accumulating mutation burden.
- In general, TSM is more prevalent than NSM.

LocHap is different from existing subclonal callers (35,41–47) in a fundamentally distinct way. LocHap provides direct evidence (e.g., examples in Figure 1) of genome mosaicism in both non-cancer and cancer cells. The units of analysis under LocHap are haplotypes, each as a scaffold of SNVs. In contrast, most subclone callers in the literature analyze allelic fractions of individual SNVs. We argue that LocHap provides a more direct view on genome mosaicism for somatic samples. The power of detecting LHVs is affected by the length of paired-end reads and coverage. In this context, it is important to note that an unexpected insert size in a paired-end read is handled by either initial choice of the value K (if it is too large) or by using a post-processing filter (if it is too small). In addition, our analysis does not include paired-end reads that are not properly mapped since these reads do not provide reliable information about LHVs.

Naturally, sequencing coverage, quality and read length affect the performance of LocHap. Deeper coverage allows LHVs with small population frequencies in the sample to be detected. Longer read length (and/or insert length) allows SNVs that are farther apart to be phased and therefore improve the chance of detecting LHVs. In our WES data our coverage was about 30× with read length 75 bps and in WGS data we have about 60× coverage with read length 100 bps. We found that our LocHap performed well in reporting LHVs under these conditions. For detail of all the bioinformatics pipelines, filters and parameters, we refer the readers to the Supplemental Data.

Our main purpose is not to identify all the LHVs in the genome. Instead, we aim to utilize existing short-read NGS data and provide a new method for detecting sample heterogeneity and mosaicism based on LHVs. Presence of LHVs itself supports mosaicism since a homogeneous human biological sample cannot harbor LHVs.

LocHap is available at http://www.compgenome.org/lochap/ for free download. A manual is provided along with the software. It is ultrafast in calling LHVs. For one WES sample with about 30× depth of coverage, whole-exome LHV calling by LocHap took about 11 s on a Macbook Pro (2.8GHZ Intel Core i7 and 16GB 1600 MHz DDR3 memory). For each WGS dataset with about 60× depth of coverage the analysis took about 47 s.

Cellular mosaicism based on LHVs would facilitate studies on heterogeneity of cell populations. Availability of NGS data allows for more powerful investigation of somatic cell subpopulations. The resolution of analysis can be at single nucleotide level, as opposed to mega-bases for microarray data. Further validation of somatic mosaicism and its relationship to aging and diseases is needed using much bigger sample sizes. Such effort could help us reveal and quantify heterogeneity in non-cancer and cancer samples, potentially affecting cancer diagnosis and prognosis.

## CONCLUSION

Through LocHap we provide a new approach to extract information of LHs from NGS data for a single sample. We found wide-spread LHVs across genome in both tumor and non-tumor samples. These results and software tools can be used for further investigation of somatic mosaicism in human samples, helping investigators to understand the frequency and genome locations of mosaic events. Thanks to the ultrafast speed of LocHap, it can be used to analyze a large number of samples using a single computer or a small cluster. The newly developed *hcf* files follow the existing format standards for *vcf* files and can be visualized in the popular tool IGV.

## Supplementary Material

SUPPLEMENTARY DATA
