# Supplementary material for: Ultra-fast local-haplotype variant calling using paired-end DNA-sequencing data reveals somatic mosaicism in tumor and normal blood samples
Source: Nucleic Acids Res. 2015 Sep 29;44(3):e25. doi: 10.1093/nar/gkv953 (PMC4756850; doi:10.1093/nar/gkv953)
Supplement: SUPPLEMENTARY DATA [file supp_44_3_e25__index.html]

Ultra-fast local-haplotype variant calling using paired-end DNA-sequencing data reveals somatic mosaicism in tumor and normal blood samples — Ultra-fast local-haplotype variant calling using paired-end DNA-sequencing data reveals somatic mosaicism in tumor and normal blood samples — SUPPLEMENTARY DATA 

# Ultra-fast local-haplotype variant calling using paired-end DNA-sequencing data reveals somatic mosaicism in tumor and normal blood samples

## SUPPLEMENTARY DATA

- SUPPLEMENTARY DATA
