# Supplementary material for: Ultra-fast local-haplotype variant calling using paired-end DNA-sequencing data reveals somatic mosaicism in tumor and normal blood samples
Source: Nucleic Acids Res. 2015 Sep 29;44(3):e25. doi: 10.1093/nar/gkv953 (PMC4756850; doi:10.1093/nar/gkv953)
Supplement: SUPPLEMENTARY DATA [file supp_gkv953_nar-01140-met-k-2015-File016.pdf]

Supplementary Data: Ultra-Fast Local-Haplotype Variant Calling Using Paired-end DNA-Sequencing Data Reveals Somatic Mosaicism in Tumor and Normal Blood Samples

Subhajit Sengupta[1], Kamalakar Gulukota[2], Yitan Zhu[1],
Carole Ober[4], Katherine Naughton[4], William Wentworth-Sheilds[4], Yuan Ji[1,3] *

MATHEMATICAL DETAIL

We show how to derive part $\mathcal{I}$ in equation (1) in the paper. Recall that the goal is to compute

$$\begin{aligned} Pr(\lambda_j = 1 \mid \boldsymbol{s}^{obs}, \boldsymbol{m}) &= Pr(\lambda_j = 1 \mid \boldsymbol{s}^{obs}, \not{\boldsymbol{m}}) \propto \underbrace{p(\boldsymbol{s}^{obs} \mid \lambda_j = 1)}_{\text{likelihood}} \underbrace{Pr(\lambda_j = 1)}_{\text{prior}} \\ &= \sum_{\boldsymbol{\lambda}_{-j} \in \mathcal{Y}_{L-1}} p(\boldsymbol{s}^{obs} \mid \lambda_j = 1, \boldsymbol{\lambda}_{-j})\, Pr(\lambda_j = 1, \boldsymbol{\lambda}_{-j}) \\ &= \sum_{\boldsymbol{\lambda}_{-j} \in \mathcal{Y}_{L-1}} \left[\prod_{i=1} \underbrace{p(\boldsymbol{s}_i^{obs} \mid \lambda_j = 1, \boldsymbol{\lambda}_{-j})}_{\mathcal{I}} \underbrace{Pr(\lambda_j = 1, \boldsymbol{\lambda}_{-j})}_{\mathcal{II}} \right]. \end{aligned} \tag{1}$$

Expanding the first term $\mathcal{I}$ of Equation (1) and augmenting the model with indicators $z_i \in \{1, \ldots, L\}$ for which $\{z_i = j\}$ is defined as the event that read i is generated from a DNA segment possessing haplotype j, we have

$$\mathcal{I} = \sum_{j'=1}^{L} p(\boldsymbol{s}_i^{obs} \mid z_i = j', \lambda_j = 1, \boldsymbol{\lambda}_{-j}) Pr(z_i = j' \mid \lambda_j = 1, \boldsymbol{\lambda}_{-j}). \tag{2}$$

Let e_{ir} be the error probability for the nucleotide called at base r on short read i. Typically e_{ir} is known from upstream analysis (e.g., in the form of *Phred quality score*). Following the notion in Ji et al. [1], we assume that the observed data s_{ir}^{obs} follows a multinomial prior

$$Pr(s_{ir}^{obs} = b_{ir} \mid z_i = j', \lambda_j = 1, \boldsymbol{\lambda}_{-j}) = (1 - e_{ir})^{I(a_{ir} = h_{jr})} \prod_{b_{ir} \neq h_{jr}} \left(\frac{e_{ir}}{3} \right)^{I(a_{ir} \neq h_{jr})}, \tag{3}$$

where $a_{ir} \in \{A, C, G, T\}$ are observed genotype of SNV r on read i, and the index set $\{a_{ir} \neq h_{jr}\}$ has three elements referring to the three nucleotides that are different from h_{jr}. We also assume that $Pr(\boldsymbol{s}_i^{obs} = \boldsymbol{b}_i) = \prod_{\{r \in \mathcal{A}_i\}} Pr(s_{ir}^{obs} = b_{ir})$. That is, the joint prior of $\boldsymbol{s}_i^{obs}$ factors over the prior (3) for the observed bases. Then (2) becomes

$$\mathcal{I} = \sum_{j'=1}^{L} \left[Pr(z_i = j' \mid \lambda_j = 1, \boldsymbol{\lambda}_{-j}) \times \prod_{r \in \mathcal{A}_{j'}(\boldsymbol{s}_i^{obs})} \left\{ (1 - e_{ir})^{I(a_{ir} = h_{j'r})} \prod_{a_{ir} \neq h_{j'r}} \frac{e_{ir}}{3} \right\} \right] \tag{4}$$

*[1]Program of Computational Genomics & Medicine, NorthShore University HealthSystem, Evanston, IL, USA. [2]Center for Molecular Medicine, NorthShore University HealthSystem, Evanston, IL, USA. [3]Department of Health Studies, University of Chicago, Chicago, IL, USA. [4]Department of Human Genetics, University of Chicago, Chicago, IL, USA. Correspondence should be addressed to Y.J. (koaeraser@gmail.com).

Lastly, we define a prior for z_i. Assume

$$Pr(z_i = j' \mid \lambda_j = 1, \boldsymbol{\lambda}_{-j}, \boldsymbol{q}_i) = c_{1i}(\boldsymbol{\lambda}) I(\lambda_{j'} = 1) \cdot \frac{1}{\sum_{\tilde{j}=1}^{L} \lambda_{\tilde{j}}}, \tag{5}$$

where $c_{1i}(\boldsymbol{\lambda})$ is a normalizing constant. The indicator $I(\lambda_{j'} = 1)$ is needed since if the haplotype j' is not present in the sample, the probability that read i is from j' should be zero.

Plugging into (2) we get the equation in the paper, i.e.,

$$\mathcal{I} = \sum_{j'=1}^{L} \left[I(\lambda_{j'} = 1) \cdot c_{1i}(\lambda) \frac{1}{\sum_{\tilde{j}=1}^{L} \lambda_{\tilde{j}}} \times \sum_{\boldsymbol{b}_i \in \{A,C,G,T\}^{w_i}} \left\{ \prod_{r \in \mathcal{A}_{j'}(\boldsymbol{s}_i^{obs}, \boldsymbol{s}_i^{mis} = \boldsymbol{b}_i)} (1 - e_{ir}) \times \prod_{r \in \mathcal{D}_{j'}(\boldsymbol{s}_i^{obs}, \boldsymbol{s}_i^{mis} = \boldsymbol{b}_i)} \frac{e_{ir}}{3} \right\} \right].$$

Additional Computational Detail

The goal is to calculate the probability in equation (6) below. Algorithm 1 in the main paper speeds up computation by taking advantage of the repeated items in the summation.

$$Pr(\lambda_j = 1 \mid \boldsymbol{s}^{obs}) \propto \sum_{\boldsymbol{\lambda}_{-j} \in \mathcal{Y}_{L-1}} \left[\underbrace{\prod_{i=1}^{N} \underbrace{Pr(\boldsymbol{s}_i^{obs} \mid \lambda_j = 1, \boldsymbol{\lambda}_{-j})}_{\mathcal{I}} \underbrace{Pr(\lambda_j = 1, \boldsymbol{\lambda}_{-j})}_{\mathcal{II}}}_{\mathcal{III}} \right] \tag{6}$$

We state Algorithm 1 again. Denote the set of indices for the event $\{\lambda_1 = 1 \mid \boldsymbol{s}^{obs}\}$ and $\{\lambda_1 = 0 \mid \boldsymbol{s}^{obs}\}$ by $\mathcal{V}_{j1}$ and $\mathcal{V}_{j0}$, respectively.

Algorithm 1 Algorithm for computing $Pr\{\lambda_j = 1 \mid \boldsymbol{s}^{obs}\} \quad \forall j = 1, 2, \ldots, L$

Index all the configurations of $\boldsymbol{\lambda}$ from 0 to $(2^L - 1)$.
Enumerate $\mathcal{V}_{j1}$ and $\mathcal{V}_{j0}$ for all $j = 1, 2, \ldots, L$.
Compute $Pr(\boldsymbol{s}^{obs} \mid C_l)$ and $Pr(C_l)$ for all $l = 0, 1, \ldots, 2^L - 1$.
for $j = 1, 2, \ldots, L$ **do**
  $P_1(j) \leftarrow \sum_{l \in \mathcal{V}_{j1}} Pr(\boldsymbol{s}^{obs} \mid C_l)\ Pr(C_l)$.
  $P_0(j) \leftarrow \sum_{l \in \mathcal{V}_{j0}} Pr(\boldsymbol{s}^{obs} \mid C_l)\ Pr(C_l)$.
  $Pr(\lambda_j = 1 \mid \boldsymbol{s}^{obs}) \leftarrow \frac{P_1(j)}{P_1(j) + P_0(j)}$.
end for

Calculation of $Pr(\boldsymbol{s}^{obs} \mid C_l)$ for all $l = 0, 1, \ldots, 2^L - 1$ in Algorithm 1 is carried out in two parts. In the first part $Pr(\boldsymbol{s}_i^{obs} \mid \lambda_j = 1, \boldsymbol{\lambda}_{-j})$ is calculated for all $i = 1, 2, \ldots, N$ (as shown by $\mathcal{I}$ in Equation (6)) and in the second part we calculate the prior term $Pr(\lambda_j = 1, \boldsymbol{\lambda}_{-j})$ as shown in $\mathcal{II}$ in Equation (6).

With the use of appropriate data structure in C++, we can find out the DNA sequences of the haplotype, $\boldsymbol{h}_j = \{h_{jr} : j = 1, 2, \ldots, L;\ r = 1, 2, \ldots, R\}$ by a linear ($\mathcal{O}(n)$) scan through all of the data. After that, for all the data $\{\boldsymbol{s_i}^{obs} : i = 1, 2, \ldots, N\}$ and all the haplotypes $\boldsymbol{h}_j$, we create a matrix D that stores a triplet $\{w_i, a_{i,j}, d_{i,j}\}$ in each cell (i, j). Recall that, w_i is the number of missing bases in

the i-th read. $a_{i,j}$ and $d_{i,j}$ denote the number of agreeing and disagreeing bases of $\boldsymbol{s}_i^{obs}$ with $\boldsymbol{h}_j$. From equation (5), let

$$T_{ij'} = Pr(z_i = j' \mid \lambda_j = 1, \boldsymbol{\lambda}_{-j}) = c_{1i}(\boldsymbol{\lambda}) I(\lambda_{j'} = 1) \cdot \frac{1}{\sum_{\tilde{j}=1}^{K} \lambda_{\tilde{j}}} \tag{7}$$

where $c_{1i}(\boldsymbol{\lambda})$ is a normalizing constant. Denoting $E_{ij'}$ by

$$E_{ij'} = \sum_{\boldsymbol{b}_i \in \{A,C,G,T\}^{w_i}} \left\{ \prod_{r \in \mathcal{A}_{j'}(\boldsymbol{s}_i^{obs}, \boldsymbol{s}_i^{mis} = \boldsymbol{b}_i)} (1 - e_{ir}) \times \prod_{r \in \mathcal{D}_{j'}(\boldsymbol{s}_i^{obs}, \boldsymbol{s}_i^{mis} = \boldsymbol{b}_i)} \frac{e_{ir}}{3} \right\}. \tag{8}$$

we have from Equations (6), (7), (8)

$$\mathcal{I} = \sum_{j'=1}^{L} \left[T_{ij'} \times E_{ij'} \right] \tag{9}$$

Note that this calculation is for one particular configuration of $\boldsymbol{\lambda}$.

We denote e_{ir} to be the error probability for the base called at locus r on short read i. Currently, for all $i = 1, 2, \ldots, N$ and for all $r = 1, 2, \ldots, R$, e_{ir} is taken as e. So after generating the matrix D, the error matrix E (given in Equation 8) is very easy to compute from D if we use a single e. Each term of $T_{ij'}$ and $E_{ij'}$ is straightforward to calculate depending on ${\boldsymbol{s}_i}^{obs}$, w_i, $\boldsymbol{h}_{j'}$ and the particular configuration of $\boldsymbol{\lambda}$. Equation (9) can be easily calculated by first doing a Hadamard matrix multiplication of T by E and then summing the elements from each row i of the resultant matrix F.

Algorithm 2 Algorithm for computing $Pr(\boldsymbol{s}_i^{obs} \mid C_l) \quad \forall l = 0, 1, \ldots, 2^L - 1$

Calculate each term $T_{ij'}$ to form the matrix T.
Calculate each term $E_{ij'}$ to form the matrix E.
Do a Hadamard matrix multiplication of T by E i.e $F \leftarrow T \circ E$.
$Pr({\boldsymbol{s}_i}^{obs} \mid C_l) \leftarrow \sum_{j=1}^{L} F(i, j)$.

The *hcf* FILE FORMAT

Each line in the *hcf* file contains information about one particular LHV segment. Below is a line in an *hcf* file from analysis of real-world data.

$##CHROM \quad POS \quad REF \quad NumSig \quad HAP\_Call \quad All\_HAP \quad DataForSample = NA12878$
$chr1 \quad 4369613, 4369623 \quad GA \quad 3 \quad GA(1.000), AA(1.000), GG(0.985) \quad GA(1.000; 0.000), AA(1.000; 0.000), GG(0.985; 0.005), AG(0.000; 0.254)$
$nSNP = 2; nTot = 90; nACGT = 75; nBlank = 15; nDisc = 0; nM0 = 41; nM1 = 34; nM2 = 15; nClus = 3;$

Same as *vcf*, an *hcf* file is a tab-delimited text file. After the initial header fields, each line in the hcf file represents a local haplotype (might not be a variant) and has seven column fields, which are explained next.

Chromosome name (CHROM), SNV positions on the chromosome (POS), nucleotides at those positions in the reference genome (REF), number of significant haplotypes (NumSig), called haplotypes (HAP-Call), all the possible haplotypes (All-HAP) and data for the sample (DataForSample=sample-name). In the "Hap-Call" field, we include the posterior probability of each haplotype variant that is considered statistically significant. The "All-HAP" field contains the posterior probability and corresponding posterior false discovery rate (FDR) for each possible haplotype. Haplotype variants in the "Hap-Call" field are generated from those in "All-HAP" by using an FDR threshold of 0.01. In the last field, a few basic statistics about the input data are given, that include total number of SNPs (nSNP), total number of reads

(nTot), number of reads having at least one entry in one of the SNP position (nACGT), number of blank reads (nBlank), number of discrepant reads (nDisc), number of reads with no missing entries, missing entries in one position or two positions or three positions (nM0, nM1, nM2, nM3, respectively) and number of clusters directly observed from the data (nClus). Note that, number of unique read groups of data that has no missing SNP defines the number of clusters. For more details refer to the *Quick Manual* document (`http://compgenome.org/lochap/code_release/QuickManual-LocHap-release-v1.0.pdf`) of LocHap software.

Summary of data for a sample: At the end of each *hcf* file a summary stating the total number of SNVs in the *vcf*, number of segments with zero significant haplotypes, one significant haplotype, two significant haplotypes and so on, are given. Below is an example.

Analyzed 6378548 Variants in Trio.VCF
Number of Segments with 0 significant haplotypes = 66654
Number of Blocks with 1 significant haplotypes = 11514
Number of Blocks with 2 significant haplotypes = 217458
Number of Blocks with 3 significant haplotypes = 18233
Number of Blocks with 4 significant haplotypes = 1205
Number of Blocks with 5 significant haplotypes = 43
Number of Blocks with 6 significant haplotypes = 2
Number of Blocks with 7 significant haplotypes = 0
Number of Blocks with 8 significant haplotypes = 0
Number of Blocks with more than 3 Variants = 192985 (Not analyzed)

ANALYSIS DETAIL

Universal setup

We analyzed the *vcf* file containing all the variant calls to identify segments with multiple proximal SNVs. Using APIs of *SAMTools*, we developed a computer program to extract nucleotides of read bases that were aligned with the SNVs using indexed *bam* files. For all the Illumina data, reads with an alignment quality score less than 30 were discarded. In addition, if a base had a Phred quality score less than 30, the called DNA sequence was ignored and a missing base M was recorded. Thus, we only kept short reads with high quality alignment and bases with high quality calling scores.

HNC data Exome sequencing data of 30 pairs of tumor and matched normal samples (total 60 samples) from patients with head and neck cancer [2] were downloaded from the Sequence Read Archive (SRA) (`http://www.ncbi.nlm.nih.gov/sra`). We extracted *fast-q* sequences from the SRA files and used BWA [3] to map short reads to the HG19 human reference genome [4]. Next, we used SAMTools [5] on each sample to generate a *bam* and a *bai* file, removing polymerase chain reaction (PCR) duplicates. We then used the realigner-target-creator and indel-realigner modules of *GATK* version 2.1.9 to refine alignments near all indels [6]. Finally, we called SNPs for all 60 samples with the UnifiedGenotyper module of *GATK* (version 2.1.9) using a minimum base quality threshold of 30 ("-mbq 30"). *GATK* caps the quality score of a base at its mapping quality and hence this also forces *GATK* to ignore any reads mapped with an alignment quality less than 30. All 60 samples were analyzed together in a single run of the UnifiedGenotyper and a single *vcf* file was generated containing all the variants across all the samples.

CEU-TRIO data The TRIO dataset was generated by whole-genome sequencing. A similar pre-processing scheme was taken to generate the *bam/bai* files and a single *vcf* file.

Validation CGI data CGI sequencing platform employs high-density DNA nanoarrays that are populated with DNA nanoballs (DNBs) and base identification is performed using a non-sequential, unchained read technology, known as combinatorial probe-anchor ligation (cPAL). CGI sequencing technology, including the general library construction process and ligation-based assay approach, the the methodology and performance of the platform are described in [7]. More information on read mapping, variation calling, read data format can be found in (`http://www.completegenomics.com/documents/DataFileFormats_Standard_Pipeline_2.5.pdf`). The data corresponding to a single genome is organized into three main directories -

- ASM – Assembly of the complete genome: variations called, coverage, and annotations.
- LIB – DNB structure for the library used in the sequencing assay.
- MAP – Reads, quality scores, and alignments to the reference genome.

The variation (*var*) file (`http://www.completegenomics.com/documents/DataFileFormats_Standard_Pipeline_2.5.pdf`) contains records for each position in the reference genome, describing whether the corresponding position was called in the CGI data, and if so, whether it is called as reference (its sequence is same as the reference genome) or variant. This is done independently for each of the two diploid alleles of the sequenced genome. The master variations file (*masterVarBeta*) is a simple, integrated report of the variant calls and annotation information produced by the CGI assembly process. The file format is derived heavily from the variations file format. The LocHap pipeline requires *bam* and *vcf* files as inputs. We generated corresponding *bam* and *vcf* files from CGI data using open source tool CGATools (`http://cgatools.sourceforge.net/docs/1.8.0/cgatools-user-guide.pdf` and `http://cgatools.sourceforge.net`) and SAMTools [5].

Model Checking

We performed some basic model checking using graphical plots to examine the model estimates against observed data. For example, for the HNC data, we present

the box plots in Fig. S1 which compares the number of inferred LHs against the number of unique read-groups mapped to all SNVs, each read-group refers to a set of reads possessing a unique genotype and mapping to all the SNVs in the segment without missing bases. For example, in Fig. 1 of the main article, the number of unique read-groups is three for both examples. Intuitively, there should be concordance between the two numbers. For example, if the number of unique read-groups is higher, there should be more inferred LHs since unique read-groups directly support the presence of the corresponding haplotypes. We see this concordance in the box-plots.

Copy number variation (CNV) analysis

Double hits of a structure mutation such as CNV and sequence mutation could falsely be explained by somatic mosaicism even if cells are homogeneous in a sample (Fig. S2). For example, a copy number gain coupled with a somatic mutation on the additional copy could lead to greater than 2 local haplotypes with short-read-based next-generation sequencing. Therefore, we further examine CNVs on LHV segments generated by LocHap. We used XHMM [8–10] for the HNC data and CNVnator [11,12] for CEU TRIO data for CNV analysis. In both examples shown in the paper, we did not find enrichment of CNVs in the regions where LHVs were present, suggesting that the haplotype variants were not associated with copy number variants. In the HNC data, we essentially found no ($< 0.01\%$) CNVs in any of the LHVs.

Additional simulation result for testing Sensitivity and Specificity

Simulation setup: We have used two different settings for the additional simulation study. In the first setting, we took a segment containing two SNVs, which had three different haplotypes. So it was a local haplotype variant (LHV) segment. In the second setting, we took a segment containing two SNVs, which had two different haplotypes. Therefore it was *not* a local haplotype variant (LHV) segment. We ran 100 simulations for each LHV segment. In each simulation, we generated a random number (between 160 and 180) of short reads that mapped to at least one SNV.

After that we used LocHap to call the number of haplotypes with their corresponding genotypes based on the observed short reads. In the first setting, we defined "sensitivity" as the ratio between the number of simulations where we successfully called three significant haplotypes with the correct genotypes and the total number of simulations and in the second setting, we defined "specificity" as the ratio between the number of simulations where we successfully called two significant haplotypes with correct genotypes and the total number of simulations.

Sensitivity Results: Simulated short reads were generated from three *true* haplotypes with proportions $0.5, 0.25$ and 0.25. We assumed at each SNV, one could observe up to two different alleles. With probability $(1 - e_b)$ we generated a genotype on the short read that is same as the true genotype of either allele and a different genotype with probability e_b. That is, e_b is the error probability for each SNV of the short read.

Recall that in the posterior inference first the inferred haplotypes were sorted according to the decreasing order of posterior probabilities and then the FDR threshold was used to select the reported haplotypes. Here we used two different values of FDR thresholds 0.01 and 0.001. With different setting of e_b we tabulated the results in Table S10. Sensitivity is worse with higher error rates e_b and gets better with a less stringent FDR threshold.

Specificity Results: We assumed now the segment has only two *true* haplotypes, with proportions 0.5 and 0.5. Here also, with different setting of e_b we report the following results in Table S11. Specificity is worse with higher error rates e_b and gets better with a more stringent FDR threshold.

Filters

LocHap includes optional post-processing filters. Despite the proposed principled and rigorous statistical inference, noise and artifacts from NGS data can still affect the quality of the estimated LHVs. We introduce an additional filtering method to remove dubious LHVs in the *hcf* files. We devise three types of multi-staged filtering schemes. First we introduce some abbreviations.

- *nHaplo* is the number of inferred haplotypes within an LHV segment.
- *nClus* is the number of unique read-groups observed directly from the short read data within an LHV segment. For example, in Fig. 1 of the main article *nClus* is 3 for both examples.
- *min-SNV-dist* is the shortest distance allowed between two successive SNVs within one segment. This will be used to remove SNVs that are too close to each other, due to potential bias caused by artifacts reported in Pickrell et al. [13].
- *short-RL* is the short-read length from NGS data.

Three different types of filters are proposed according to their stringency levels - type I, type II, type III. Among all three filter types, type I is the most conservative and type III is the least. Here, a conservative filter would remove more estimated LHVs in an *hcf* file. The filters remove LHV segments from the *hcf* files based on the following criteria. Metaphorically, we consider the filtering a process of revealing a portrait of an elephant. Without knowing which animal is on the portrait, Type I filter only reveals the tail of the animal, Type II filter reveals legs and tail, and Type III reveals the whole elephant.

- Common to all three types is a Fisher's exact test for testing the strand bias [14, 15]. An LHV segment is removed if the if short reads show strand bias based on a significance level of 0.05. Also, we require that at least one read is mapped to each strand.
- Type I (tail): { *nHaplo* > 2 and *nClus* > 2 and *min-SNP-dist* = *short-RL* }
- Type II (leg): { *nHaplo* > 2 and *min-SNP-dist* = *short-RL* }
- Type III (elephant): { *nHaplo* > 2 and *min-SNP-dist* = *short-RL / 2* }

In addition, the following filtering steps are applied to all three types of filters based on discussion in the literature [15–17]. These filters aim at removing short reads, not segments, that have poor quality.

- If on either end of a pair-end read, an Indel is called along with an SNV, the read is removed. Typically co-occurrence of different mutations on the same end of a read is caused by artifact and noise in base calling.
- Any SNVs are removed if they reside on a region within 10% of the read length from the end of any short read.

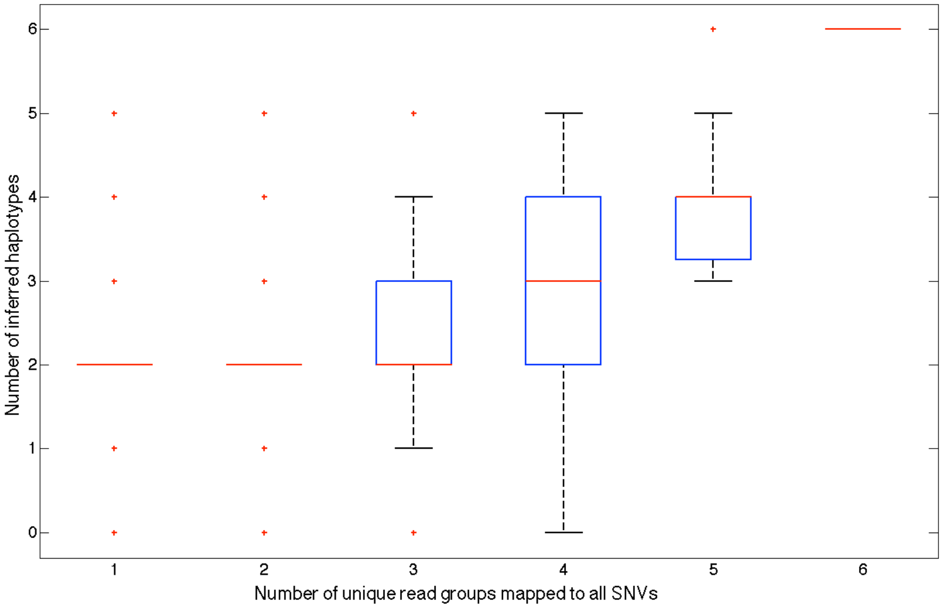

Figure S1: Model Checking. Boxplots of numbers of inferred haplotypes (vertical axis) for different numbers of unique read-groups (horizontal axis)

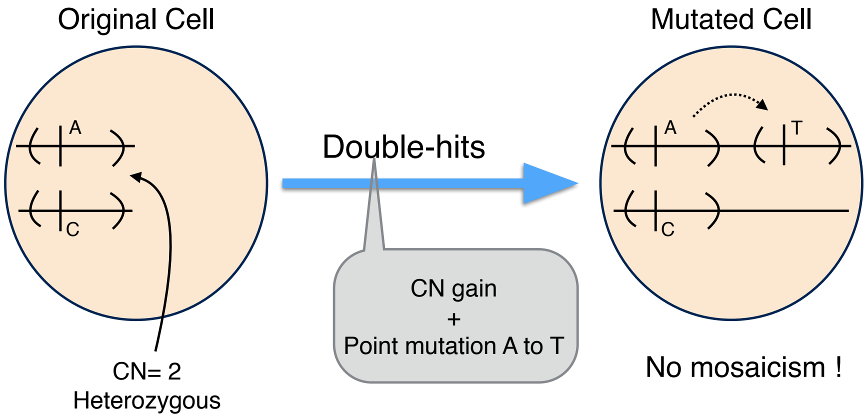

Figure S2: Illustration of a hypothetical event of “Double-hits”. A copy number gain occurs and on the new copy, a point mutation replaces an “A” with a ‘T”. When all three copies are sequenced, potentially three different alleles bearing “A”, “T” and “C” could be observed. Without accounting for copy number gains, somatic mosaicism could be falsely inferred.

Tabulated Posterior Probabilities for Simulated Data

Sequences	Frequency
AA	9
A –	23
GA	8
GG	7
G –	4
– A	42
– G	19

Table S1: A simulation data set for the calibration of e_{ir}. Short reads with only two bases are generated and their sequences and frequencies are shown.

Sequence	Post. Prob.	FDR
GA	1.0000000	0.0000000
AC	0.9999428	0.0000286
GC	0.9997732	0.0000946
AA	0.0049901	0.2488235

Table S2: Simulation scenario 1. Shown are possible haplotype sequences with corresponding posterior probabilities ξ_j and estimated posterior expected FDR.

Sequence	Post. Prob.	FDR
GG	0.5474743	0.4525257
AG	0.5425159	0.4550049
GA	0.5179061	0.4640346
AA	0.4887007	0.4758507

Table S3: Simulation scenario 2. Shown are possible haplotype sequences with corresponding posterior probabilities ξ_j and estimated posterior expected FDR.

Sequence	Post. Prob.	FDR
AGA	0.9999828	0.0000171
AGC	0.9999828	0.0000171
GAC	0.9989948	0.0003464
AAC	0.0122725	0.2471917
GGC	0.0122725	0.3952988
GAA	0.0122641	0.4940383
AAA	0.0119430	0.5646124
GGA	0.0119430	0.6175430

Table S4: Simulation scenario 3. Shown are possible haplotype sequences with corresponding posterior probabilities ξ_j and estimated posterior expected FDR.

Sequence	Post. Prob.	FDR
AAA	0.9470780	0.0529220
AAT	0.9470780	0.0529220
ACA	0.9470780	0.0529220
ACT	0.9470780	0.0529220
GAA	0.9470780	0.0529220
GAT	0.9470780	0.0529220
GCA	0.9470780	0.0529220
GCT	0.9470780	0.0529220

Table S5: Simulation scenario 4. Shown are possible haplotype sequences with corresponding posterior probabilities ξ_j and estimated posterior expected FDR.

Sequence	Post. Prob.	FDR
AAA	0.9999999	0.0000000
AAT	0.9999999	0.0000000
ACA	0.9999999	0.0000000
ACT	0.9503068	0.0298116
GAA	0.9523648	0.0186603
GAT	0.9522508	0.0235084
GCA	0.9522508	0.0269714
GCT	0.9543334	0.0114166

Table S6: Simulation scenario 5. Shown are possible haplotype sequences with corresponding posterior probabilities ξ_j and estimated posterior expected FDR.

Sequence	Post. Prob.	FDR
GA	1.0000000	0.0000000
AC	1.0000000	0.0000000
GC	1.0000000	0.0000000
AA	0.0000020	0.2499994

Table S7: Simulation scenario 6. Shown are possible haplotype sequences with corresponding posterior probabilities ξ_j and estimated posterior expected FDR.

Sequence	Post. Prob.	FDR
GG	1.0000000	0.0000000
GC	1.0000000	0.0000000
AG	1.0000000	0.0000000
AC	0.0000000	0.2499999

Table S8: Simulation scenario 7. Shown are possible haplotype sequences with corresponding posterior probabilities ξ_j and estimated posterior expected FDR.

Sequence	Post. Prob.	FDR
TT	1.0000000	0.0000000
CG	1.0000000	0.0000000
CT	1.0000000	0.0000000
TG	0.0000000	0.2499999

Table S9: Simulation scenario 8. Shown are possible haplotype sequences with corresponding posterior probabilities ξ_j and estimated posterior expected FDR.

e_b	Sensitivity (with FDR=0.01)	Sensitivity (with FDR=0.001)
0.01	1.00	1.00
0.05	0.87	0.86
0.10	0.57	0.55

Table S10: Sensitivity analysis for simulated data from a segment with two SNVs and three *true* haplotypes in the sample.

e_b	Specificity (with FDR=0.01)	Specificity (with FDR=0.001)
0.01	1.00	1.00
0.05	0.82	0.86
0.10	0.26	0.35

Table S11: Specificity analysis for simulated data from a segment with two SNVs and only two *true* haplotypes in the sample.

References

[1] Yuan Ji, Yanxun Xu, Qiong Zhang, Kam-Wah Tsui, Yuan Yuan, Clift Norris Jr, Shoudan Liang, and Han Liang. BM-Map: Bayesian Mapping of Multireads for Next-Generation Sequencing Data. *Biometrics*, 67(4):1215–1224, 2011.

[2] Nicolas Stransky, Ann Marie Egloff, Aaron D Tward, Aleksandar D Kostic, Kristian Cibulskis, Andrey Sivachenko, Gregory V Kryukov, Michael S Lawrence, Carrie Sougnez, Aaron McKenna, et al. The mutational landscape of head and neck squamous cell carcinoma. *Science*, 333(6046):1157–1160, 2011.

[3] Heng Li and Richard Durbin. Fast and accurate short read alignment with Burrows–Wheeler transform. *Bioinformatics*, 25(14):1754–1760, 2009.

[4] Deanna M Church, Valerie A Schneider, Tina Graves, Katherine Auger, Fiona Cunningham, Nathan Bouk, Hsiu-Chuan Chen, Richa Agarwala, William M McLaren, Graham RS Ritchie, et al. Modernizing reference genome assemblies. *PLoS biology*, 9(7):e1001091, 2011.

[5] Heng Li, Bob Handsaker, Alec Wysoker, Tim Fennell, Jue Ruan, Nils Homer, Gabor Marth, Goncalo Abecasis, Richard Durbin, et al. The sequence alignment/map format and SAMtools. *Bioinformatics*, 25(16):2078–2079, 2009.

[6] Aaron McKenna, Matthew Hanna, Eric Banks, Andrey Sivachenko, Kristian Cibulskis, Andrew Kernytsky, Kiran Garimella, David Altshuler, Stacey Gabriel, Mark Daly, et al. The Genome Analysis Toolkit: a MapReduce framework for analyzing next-generation DNA sequencing data. *Genome research*, 20(9):1297–1303, 2010.

[7] Radoje Drmanac, Andrew B Sparks, Matthew J Callow, Aaron L Halpern, Norman L Burns, Bahram G Kermani, Paolo Carnevali, Igor Nazarenko, Geoffrey B Nilsen, George Yeung, et al. Human genome sequencing using unchained base reads on self-assembling dna nanoarrays. *Science*, 327(5961):78–81, 2010.

[8] Menachem Fromer, Jennifer L Moran, Kimberly Chambert, Eric Banks, Sarah E Bergen, Douglas M Ruderfer, Robert E Handsaker, Steven A McCarroll, Michael C O?Donovan, Michael J Owen, et al. Discovery and statistical genotyping of copy-number variation from whole-exome sequencing depth. *The American Journal of Human Genetics*, 91(4):597–607, 2012.

[9] Christopher S Poultney, Arthur P Goldberg, Elodie Drapeau, Yan Kou, Hala Harony-Nicolas, Yuji Kajiwara, Silvia De Rubeis, Simon Durand, Christine Stevens, Karola Rehnström, et al. Identification of small exonic CNV from whole-exome sequence data and application to autism spectrum disorder. *The American Journal of Human Genetics*, 93(4):607–619, 2013.

[10] Menachem Fromer and Shaun M Purcell. Using XHMM Software to Detect Copy Number Variation in Whole-Exome Sequencing Data. *Current Protocols in Human Genetics*, pages 7–23, 2014.

[11] Ryan E Mills, Klaudia Walter, Chip Stewart, Robert E Handsaker, Ken Chen, Can Alkan, Alexej Abyzov, Seungtai Chris Yoon, Kai Ye, R Keira Cheetham, et al. Mapping copy number variation by population-scale genome sequencing. *Nature*, 470(7332):59–65, 2011.

[12] Alexej Abyzov, Alexander E Urban, Michael Snyder, and Mark Gerstein. CNVnator: an approach to discover, genotype, and characterize typical and atypical CNVs from family and population genome sequencing. *Genome research*, 21(6):974–984, 2011.

[13] Joseph K. Pickrell, Yoav Gilad, and Jonathan K. Pritchard. Comment on “Widespread RNA and DNA Sequence Differences in the Human Transcriptome”. *Science*, 335(6074):1302, 2012.

[14] Yan Guo, Jiang Li, Chung-I Li, Jirong Long, David C Samuels, and Yu Shyr. The effect of strand bias in Illumina short-read sequencing data. *BMC genomics*, 13(1):666, 2012.

[15] Tongjun Gu, Frank W Buaas, Allen K Simons, Cheryl L Ackert-Bicknell, Robert E Braun, and Matthew A Hibbs. Canonical A-to-I and C-to-U RNA editing is enriched at 3′ UTRs and microRNA target sites in multiple mouse tissues. *PloS one*, 7(3):e33720, 2012.

[16] Frazer Meacham, Dario Boffelli, Joseph Dhahbi, David IK Martin, Meromit Singer, and Lior Pachter. Identification and correction of systematic error in high-throughput sequence data. *BMC bioinformatics*, 12(1):451, 2011.

[17] Heng Li. Towards better understanding of artifacts in variant calling from high-coverage samples. *Bioinformatics*, 30:2843–2851, 2014.
